# Microwave-assisted commercial copper-catalyzed aerobic oxidative synthesis of AChE quinazolinone inhibitors under solvent free conditions†

**DOI:** 10.1039/d3ra05739a

**Published:** 2023-09-18

**Authors:** Jira Jongcharoenkamol, Prakansi Naksing, Nattayaporn Nimnuan, Thishana Singh, Jaruwan Chatwichien, Prapapan Temkitthawon, Chanitsara Sriwattanawarunyoo, Vachira Choommongkol, Puttinan Meepowpan, Sutthichat Kerdphon

**Affiliations:** a Department of Pharmaceutical Chemistry and Pharmacognosy, Faculty of Pharmaceutical Science, Naresuan University Phitsanulok 65000 Thailand; b Center of Excellence in Cannabis Research, Faculty of Pharmaceutical Sciences, Naresuan University Phitsanulok 65000 Thailand sutthichatk@nu.ac.th; c Department of Chemistry, Faculty of Science, Naresuan University Phitsanulok 65000 Thailand; d School of Chemistry and Physics, University of Kwazulu-Natal Private Bag X54001 Durban 4000 South Africa; e Program in Chemical Sciences, Chulabhorn Graduate Institute, Chulabhorn Royal Academy Bangkok 10210 Thailand; f Department of Chemistry, Faculty of Science, Maejo University Chiang Mai 50290 Thailand; g Department of Chemistry, Faculty of Science, Chiang Mai University Chiang Mai 50200 Thailand; h Center of Excellence in Material Science and Technology, Chiang Mai University Chiang Mai 50200 Thailand; i Center of Excellence in Petroleum, Petrochemicals and Advanced Materials, Faculty of Science, Naresuan University Phitsanulok 65000 Thailand

## Abstract

A facile and green one-pot synthesis of AChE quinazolinone inhibitors was developed using microwave irradiation under solvent free conditions. Quinazolinones were synthesized from 2-aminobenzamide derivatives and various alcohols such as benzyl alcohol derivatives and butanol using economical commercially available copper as a catalyst in the presence of base, Cs_2_CO_3_. The desired products were achieved in moderate to high yields with up to 92% isolated yield. These quinazolinone products were then evaluated for acetylcholinesterase inhibition so that they can be developed as promising anti-acetylcholinesterase agents.

## Abstract 2

A facile and green one-pot synthesis of AChE quinazolinone inhibitors was developed using microwave irradiation under solvent free conditions.

## Graphical abstract

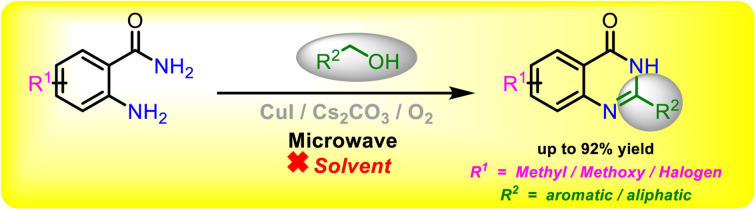

## Introduction

Quinazolinone is a privileged moiety that is found in many natural products and synthetic compounds. Most of them exhibit bioactivity properties such as antifungal, antimicrobial, antihyperglycemic, antiinflammation, anticancer, anticonvulsant as well as acetylcholinesterase (AchE) inhibition. Acetylcholine is a neurotransmitter that has many functions in the human body. This neurotransmitter is produced from presynaptic neurons. It is destroyed by AchE which breaks acetylcholine into acetic acid and choline. An excess of AchE leads to diseases including Alzheimer's and myasthenia gravis.^1^ Inhibition of AChE results in an increased concentration of acetylcholine. For the past decade, many different compounds have been discovered as well as developed to inhibit AchE enzyme including compounds containing a quinazoline core structure.^2^

Quinazolinone can be synthesized using various starting materials and different methods for example, reaction of 2-bromobenzaldehydes with acetamidine hydrochloride using copper-catalyzed reaction;^3^ 2-bromobenzamides with formamide catalyzed by CuI and 4-hydroxy-l-proline as a ligand.^4^ Also, 2-aminobenzamide with alcohols were very popular starting materials that were used under different developed methods.^5–12^ Generally, quinazolinones are synthesized through alcohol oxidation, nucleophilic addition, and cyclization. These steps consume much energy and have long reaction times. Therefore, green chemistry has been used in organic synthesis to reduce energy consumption and toxicity of either solvents or reagents in order to be more cost effective. Organometallics were also employed in a one-pot procedure to synthesize quinazolinones with high to excellent yields. Benzyl alcohols reacted with 2-aminobenzamide to give the corresponding quinazolinone in up to 85% yield under these reaction conditions: Ru(PPh_3_)_3_(CO)(H)_2_ as a catalyst and toluene at 115 °C for 14 hours.^13^ Nickel-catalyst was also used in the reaction at 100 °C and furnished the desired product in up to 90% yield.^14^ Moreover, it is reported that commercial, easy to handle copper catalysts that have copper hydroxide supported on manganese oxide octahedral molecular sieves (Cu(OH)X/OMS-2), Cu(OAc)_2_ and CuI promotes quinazolinone synthesis at 80–100 °C for 12–24 hours giving the product in up to 95% yield.^15–17^ In 2016, the Li group developed a method using microwave-assisted synthesis at 130 °C for 2 hours in methanol. They reported a starting material and solvent with [Cp*Ir(2,2′-bpyO)(H_2_O)] as a catalyst.^18^ Metal catalysts or microwave-assisted synthesis can reduce the number of reaction steps and or reaction time.^19^ However, organic solvents and specialized catalysts are still required.

Recently, our group developed a method^20,21^ for copper catalyzed one-pot quinazolinone synthesis. We also investigated their anti-inflammatory effects and anti-acetylcholinesterase activities. Hence, the focus of this investigation was on microwave-assisted commercial copper-catalyzed quinazolinone derivatives synthesized in one step under solvent-free conditions. The new method was environmentally friendly; had a short reaction time; was simple to use; and used a less toxic solvent. Moreover, synthesized quinazolinones would be screened for their ability to inhibit acetylcholinesterase. Outstanding compounds will be hit compounds for the continued development of AChE inhibitors.

## Results and discussion

For the optimization of the reaction conditions, 2-aminobenzamide and benzyl alcohol were chosen as the model substrates. 2-Aminobenzamide reacted with 5.0 equiv. of benzyl alcohol in the presence of 0.5 equiv. of Cs_2_CO_3_ base and 20 mol% of various commercially available Cu catalysts under O_2_ atmosphere at 110 °C for 1 h in a microwave reactor (Table 1, entry 1–5). CuI produced the targeted quinazolinone 3a with the highest yield of 53% (Table 1, entry 2). When Cs_2_CO_3_ base was increased to 1.0 equiv., the desired quinazolinone yield improved to 72% (Table 1, entry 5). Base screening with 20 mol% of CuI at 110 °C was investigated. When the base was changed from Cs_2_CO_3_ to K_2_CO_3_, the yield of the desired product 3a decreased to 19% (Table 1, entry 6). The hydroxide bases such as NaOH and KOH did not improve the yield of the target product. A yield of 71% and 69% was observed, respectively (Table 1, entries 7 and 8). A scale-up of the reaction using 0.5 mmol of 2-aminobenzamide with 5.0 equiv. of benzyl alcohol, 1.5 equiv. of Cs_2_CO_3_ in the presence of CuI (20 mol%) for 2 and 3 h decreased the isolated yield to 64% and 62%, respectively (Table 1, entries 9 and 10). When the temperature was increased to 130 °C for 1 h, the yield of target quinazolinone product was insignificantly improved (Table 1, entry 11). Surprisingly, an increase in the reaction time to 2 h at 130 °C, resulted in full conversion of 90% isolated yield of 3a (Table 1, entry 12). When the amount of benzyl alcohol was varied and either CuI or Cs_2_CO_3_ base was decreased, it gave a lower yield of the product (Table 1, entries 13–15). The results from entries 16–18 confirmed the importance of copper catalyst, Cs_3_CO_3_ base and O_2_ gas in order to form the product in high yield.

**Table 1:** Optimized reaction condition for commercially available Cu-catalyst under microwave radiation

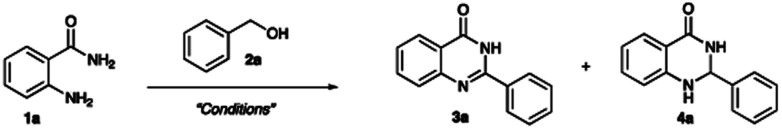									
Entry	1a (mmol)	2a (e.q.)	Catalyst (mol%)	Base (e.q.)	O_2_	Temp (°C) microwave radiation	Time (h)	Yield 3a^a^ (%)	Yield 4a^a^ (%)
1	0.2	5.0	Cu(OAc)_2_·H_2_O (20)	Cs_2_CO_3_ (0.5)	O_2_	110	1	29	1
2	0.2	5.0	CuI (20)	Cs_2_CO_3_ (0.5)	O_2_	110	1	53	1.5
3	0.2	5.0	CuBr_2_ (20)	Cs_2_CO_3_ (0.5)	O_2_	110	1	39	1
4	0.2	5.0	CuCl_2_ (20)	Cs_2_CO_3_ (0.5)	O_2_	110	1	42	0.4
5	0.2	5.0	CuI (20)	Cs_2_CO_3_ (1.0)	O_2_	110	1	72	0.4
6	0.2	5.0	CuI (20)	K_2_CO_3_ (1.0)	O_2_	110	1	19	0.9
7	0.2	5.0	CuI (20)	NaOH (1.0)	O_2_	110	1	71	0.7
8	0.2	5.0	CuI (20)	KOH (1.0)	O_2_	110	1	69	0.6
9	0.5	5.0	CuI (20)	Cs_2_CO_3_ (1.5)	O_2_	110	2	64^b^	—
10	0.5	5.0	CuI (20)	Cs_2_CO_3_ (1.5)	O_2_	110	3	62^b^	—
11	0.5	5.0	CuI (20)	Cs_2_CO_3_ (1.5)	O_2_	130	1	66^b^	—
12	0.5	5.0	CuI (20)	Cs_2_CO_3_ (1.5)	O_2_	130	2	90^b^	—
13	0.5	2.5	CuI (20)	Cs_2_CO_3_ (1.5)	O_2_	130	2	60^b^	—
14	0.5	5.0	CuI (10)	Cs_2_CO_3_ (1.5)	O_2_	130	2	56^b^	—
15	0.5	5.0	CuI (20)	Cs_2_CO_3_ (1.0)	O_2_	130	2	64^b^	—
16	0.5	5.0	No	Cs_2_CO_3_ (1.5)	O_2_	130	2	44^b^	—
17	0.5	5.0	CuI (20)	No	O_2_	130	2	n.d.	—
18	0.5	5.0	CuI (20)	Cs_2_CO_3_ (1.5)	No	130	2	62^b^	—

a NMR yield using 1,3,5-trimethoxybenzene as internal standard.

b Isolated yield.

Using the optimized reaction conditions, 2-aminobezamide derivatives were evaluated as substrates in the reaction (Table 2). The model substrate afforded the target product in a high isolated yield of 90% (Table 2, entry 1). The effect of varying substituents on the aromatic ring of various 2-aminobenzamide were investigated. 2-Aminobenzamide bearing electron-donating methyl or methoxy on the aromatic ring led to decreased percentage yields of 56% and 40%, respectively (Table 2, entries 2 and 3). However, the substrates with electron-withdrawing fluorine or chlorine substituents at the *para* position reacted successfully with benzyl alcohol to give the desired product in moderate to high yield of 79% and 46%, respectively (Table 2, entries 4 and 5). Halogens such as fluorine, chlorine, or bromine was used as a substituent at the *para* position with the amide group. In these cases, the nucleophilicity of the substrates was decreased resulting in the target products with 38%, 62% and 14% isolated yields, respectively (Table 2, entries 6–8). This catalytic system was also evaluated in the reaction of 2-aminosulfonamide and 2-aminonicotinamide and the moderate yield of the desired products were afforded (Table 2, entries 9 and 10).

**Table 2:** Synthesis of quinazolinone derivatives from 2-aminobenzamide derivatives and benzyl alcohol using microwave irradiation under solvent-free conditions^a^

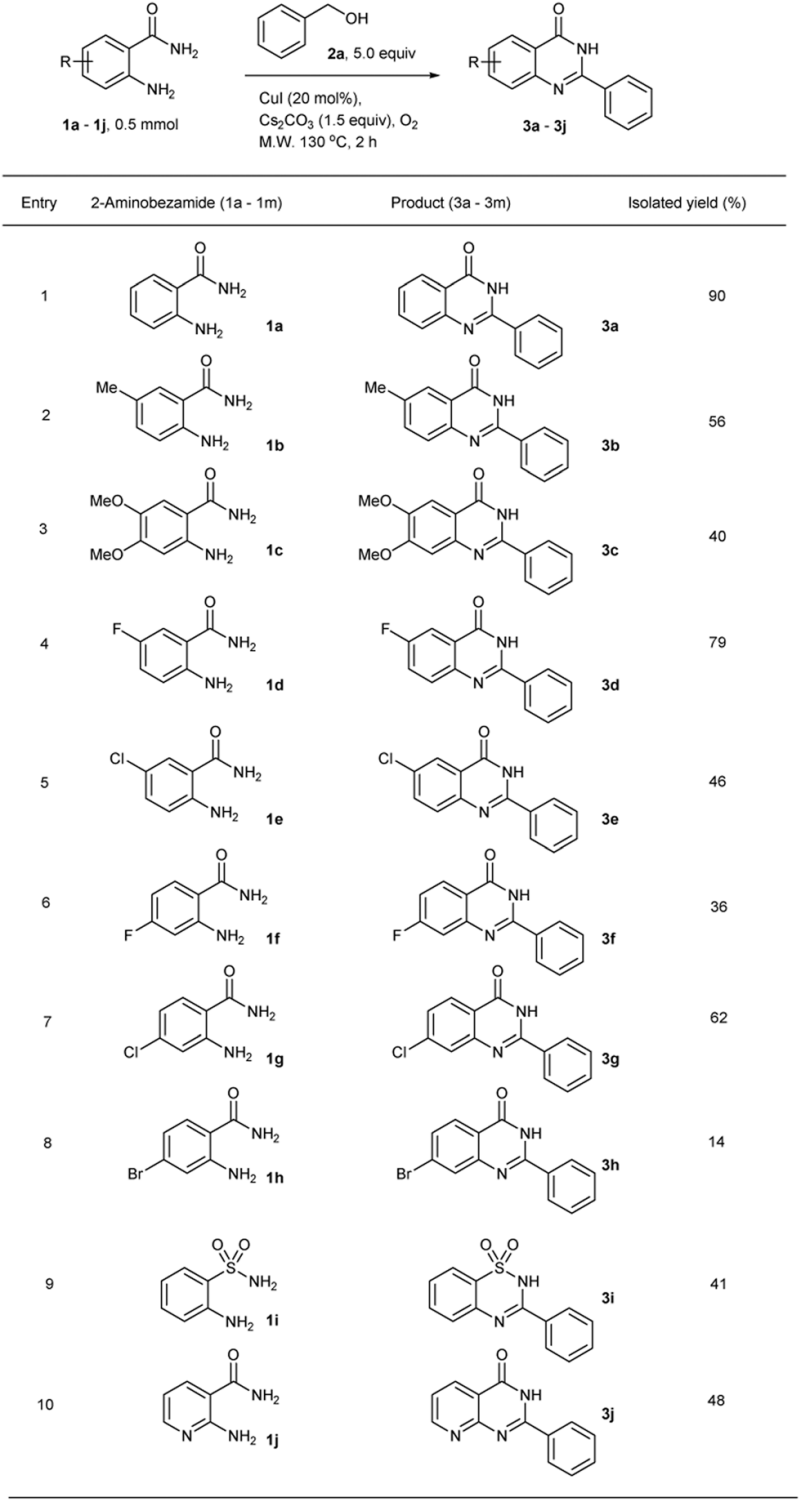

a Reaction conditions: 2-aminobenzamides (1a–1j, 0.5 mmol), benzyl alcohol (2a, 5.0 equiv.), CuI (20 mol%), Cs_2_CO_3_ (1.5 equiv.), under O_2_ atmosphere, microwave 130 °C, 2 h.

The substrate scope for benzyl alcohol derivatives and butanol were also examined (Table 3). The reaction was successful and afforded the desired products in moderate to high isolated yields. Benzyl alcohols bearing electron-donating groups such as methyl or methoxy gave the target products in up to 83% isolated yield (Table 3, entries 1–4). The effect of halogen substituents were also investigated for this reaction. Benzyl alcohol bearing the fluorine substituent at the *para* position on the aromatic ring gave the quinazolinone product in moderate yield (Table 3, entry 5). However, for the chlorine and bromine substituents on the benzyl alcohol substrate which are the solid compounds, although the desired products were obtained, the crude mixtures could not be purified (Table 3, entries 6 and 7). In these cases, the amide substrates might have a solubility problem in the alcohol reagents. Surprisingly, 73% and 92% isolated yields were obtained when *m*-trifluoromethyl benzyl alcohol and *n*-butanol were used as alkylating reagents (Table 3, entries 8 and 9). Cinnamyl alcohol was also investigated as a substrate to afford the product in 55% isolated yield (Table 3, entry 10). Then the reaction was scaled up to 300 mg (2.2 mmol) of 2-aminobenzamide which was about 5 times larger than the optimal condition. The desired product was obtained in only 30% isolated yield. In this case, the size of MW vessel which was 13 × 100 mm as the largest Duran® Culture Tube with PBT screw caps for MW vessel might affect the yield because the reaction mixture level was high. Thus, stirring was not good as lower level in the optimal condition.

**Table 3:** Synthesis of quinazolinone derivatives from 2-aminobenzamide and alcohols using microwave irradiation under solvent-free conditions^a^

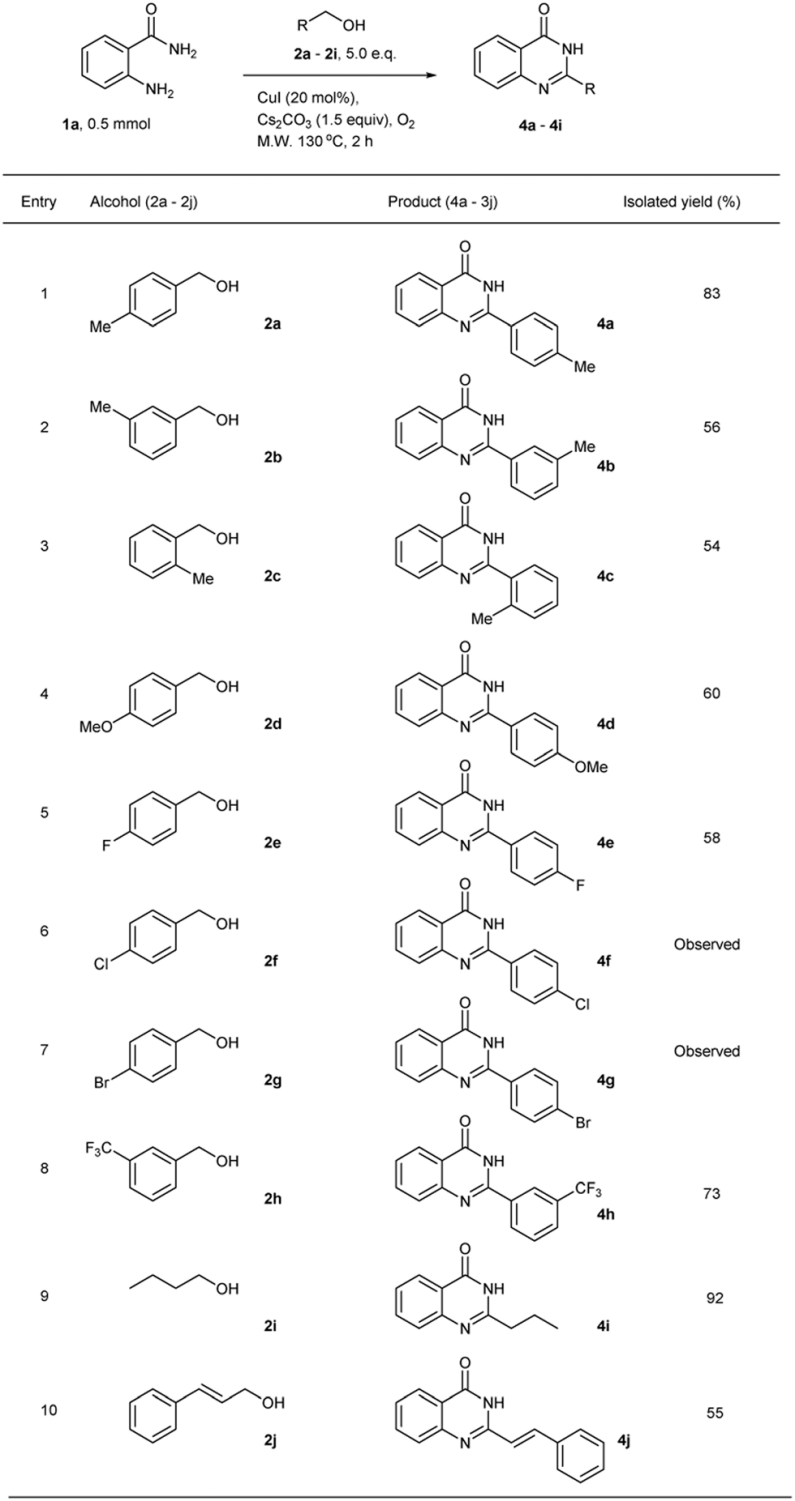

a Reaction conditions: 2-aminobenzamides (1a, 0.5 mmol), alcohol (2a–2j, 5.0 equiv.), CuI (20 mol%), Cs_2_CO_3_ (1.5 equiv.), under O_2_ atmosphere, microwave 130 °C, 2 h.

**Scheme 1:**
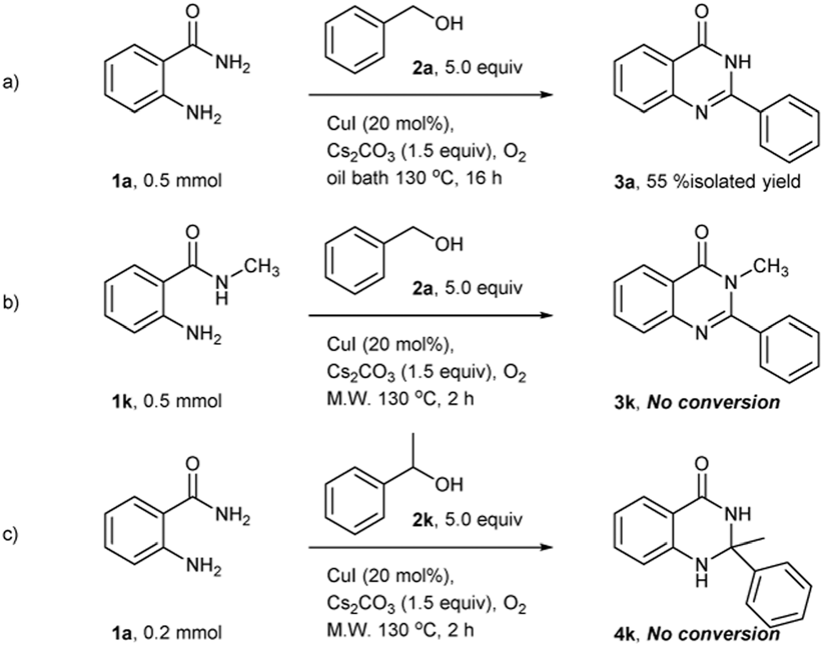
(a) The reaction was carried out with optimal condition under heating condition. (b) Using the secondary amide as a substate. (c) Using the secondary alcohol as a substrate.

When the reaction was carried out with optimal condition under heating condition using oil bath for 16 hours, the desired quinazolinone products was obtained in only 55% isolated yield. The result suggests that microwave radiation helps to increase the reactivity of this reaction (Scheme 1a). To further investigate the substrate scope, the secondary amide and secondary alcohol were used as the substrates. The target products were not obtained. It might have a steric effect in the imine formation step and the cyclization step. The crude reactions were observed only benzaldehyde and acetophenone, respectively (Scheme 1b and c).

**Scheme 2:**
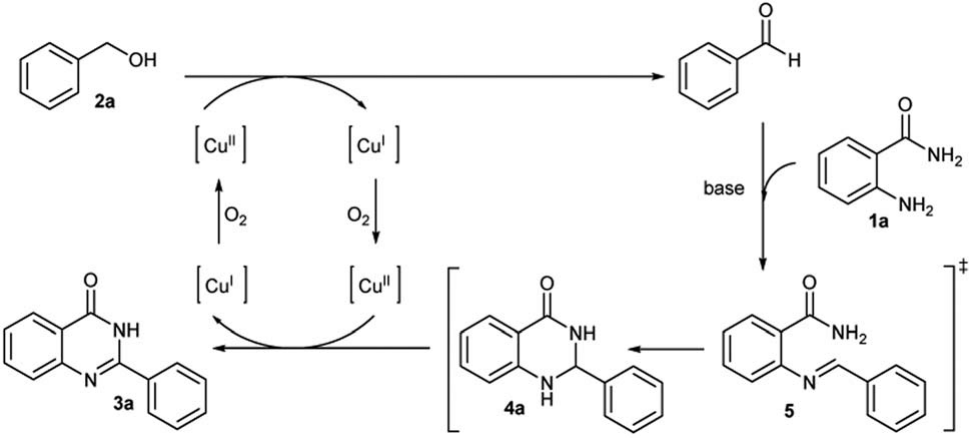
Proposed reaction mechanism.

**Scheme 3:**
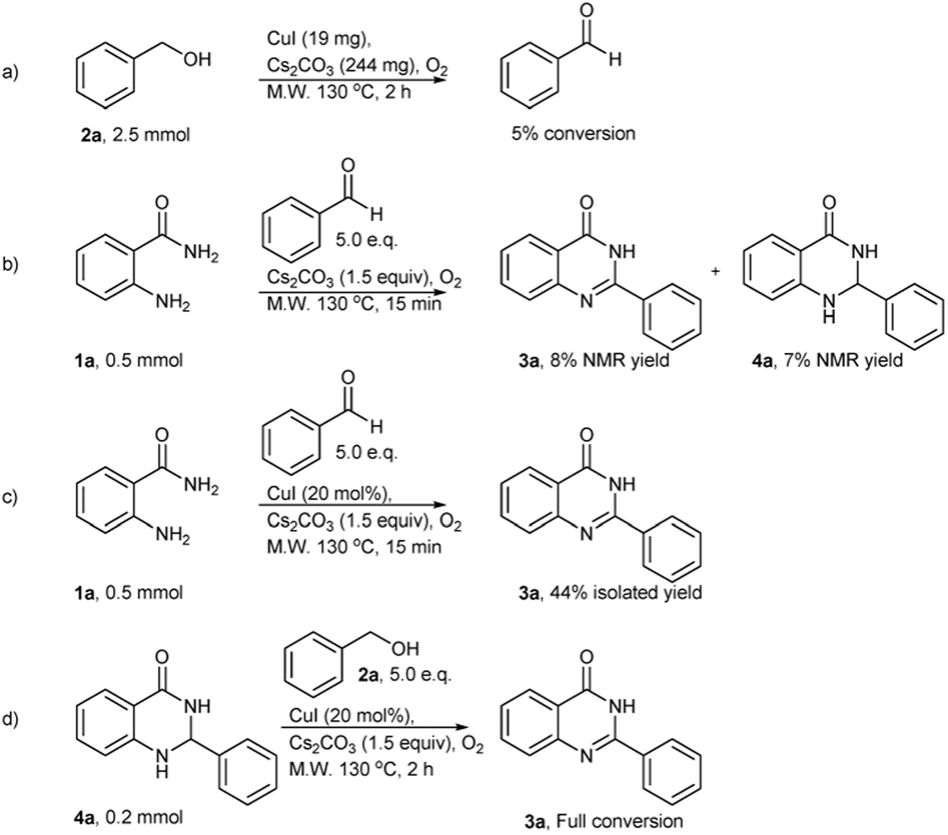
Control experiments for the proposed mechanism.

The possible mechanism of this reaction was depicted in Scheme 2. It begins with the oxidation of benzyl alcohol to benzaldehyde using the copper catalyzed system. Then benzaldehyde reacts with 2-aminobenzamide in the presence of Cs_2_CO_3_ base to give the imine intermediate 5. This is followed by cyclization to afford dihydroquinazolinone 4a. In the last step, compound 4a is oxidized by the copper catalyst to yield the target quinazolinone product 3a. This proposed mechanism was confirmed by control experiments shown in Scheme 3. The reaction was carried out in the microwave for 2 hours using the optimal conditions but without 2-aminobenzamide. The reaction contained by 4 mol% of CuI produced only benzaldehyde as the product in 5% conversion. Corresponding result confirmed the first step of the proposed mechanism (Scheme 3a).

To prove that benzaldehyde reacts with 2-aminobenzamide and is followed by the cyclization step, then the final product is formed by the help of copper catalyst. The reaction was carried out without the copper catalyst for 15 minutes. Only 8% and 7% yields of 3a and 4a were observed, respectively (Scheme 3b). The reaction was also carried out using optimal conditions with benzaldehyde instead of benzyl alcohol for 15 minutes and afforded quinazolinone product 3a in 44% isolated yield (Scheme 3c). These results suggest that the copper catalyst may be involved in the imine step formation followed by cyclization. The oxidation of compound 4a to product 3a in the last step was confirmed and intermediate 4a was used as the substrate under optimal conditions. Full conversion of quinazolinone product 3a was observed (Scheme 3d).

The synthesized quinazolinone products were evaluated to investigate inhibition of acetylcholinesterase (AChE) using the modified Ellman's spectrophotometric method as shown in Table 4.^22^ The inhibition screening of AChE showed that quinazolinones 3a and 3f containing fluorine substituent at *para* position with a carbonyl group, were the most active compounds. The inhibition percentages were calculated as 49.46% and 58.25% at 50 μM concentration for 3a and 3f, respectively. Other quinazolinones that were synthesized from 2-aminobenzamide derivatives and alcohols showed weak activity and had inhibition values of less than 45%. However, these results do indicate that these quinazolinones can be developed into anti-acetylchloinesterase agents for further study.

**Table 4:** % AChE inhibition of the quinazolinones (0.05 mM)^a^

Comp.	AChE inhibition (%)	Comp.	AChE inhibition (%)
3a	**49.46 ± 4.28**	4a	35.10 ± 7.30
3b	38.02 ± 7.94	4b	38.84 ± 3.50
3c	37.57 ± 2.48	4c	19.44 ± 0.65
3d	42.04 ± 7.92	4d	21.70 ± 8.93
3e	34.95 ± 6.85	4e	26.22 ± 6.38
3f	**58.25 ± 2.63**	4h	11.26 ± 5.01
3g	24.04 ± 3.67	4i	22 25 ± 8.42
3i	13.81 ± 1.46	4j	8.01 ± 3.17
3j	20.34 ± 1.85		

a Galanthamine was used as positive control for AChE inhibition (IC_50_ = 1.50 ± 0.15 μM).

## Conclusions

In summary, we have successfully developed a facile and environmentally friendly method for the one pot synthesis of quinazolinone and their derivatives. A variety of 2-aminobenzamide derivatives successfully reacted with benzyl alcohol under microwave radiation and solvent free conditions for 2 h to afford the desired products in moderate to high isolated yields of up to 90%. Different types of alcohols also reacted with 2-aminobenzamide and afforded the quinazolinone products in moderate to high isolated yields. Notably, aliphatic alcohol, butanol furnished the product with the highest isolated yield of 92% under this reaction condition. In the evaluation of these quinazolinone products for acetylchlolinesterase inhibitory activity, it was found that up to 58.25% inhibition at 50 μM is possible.

## Author contributions

S. K. and J. J. wrote the manuscript and ESI† with input from all the authors. S. K. undertook part of optimization conditions and mechanistic studies. S. K., J. J., P. N., N. N. and C. S. carried out all the syntheses of all substrate scope analysis. The AChE-inhibitor study was conducted by J. J. and P. T. The paper was proofread by T. S., J. C., C. S. and P. M. The NMR experiment was performed by J. C., V. C. and P. M. This project was designed and conceived by S. K.

## Conflicts of interest

There are no conflicts of interest to declare.

## Supplementary Material

RA-013-D3RA05739A-s001
