# Supplementary material for: Microwave-assisted commercial copper-catalyzed aerobic oxidative synthesis of AChE quinazolinone inhibitors under solvent free conditions
Source: RSC Adv. 2023 Sep 18;13(39):27657–62. doi: 10.1039/d3ra05739a (PMC10506383; doi:10.1039/d3ra05739a)
Supplement: RA-013-D3RA05739A-s001 [file RA-013-D3RA05739A-s001.pdf]

Electronic Supplementary Material (ESI) for RSC Advances.
This journal is © The Royal Society of Chemistry 2023

Supporting information for: Microwave-Assisted Commercial Copper-Catalyzed Aerobic Oxidative Synthesis of AChE Quinazolinone Inhibitors under Solvent Free Conditions

Jira Jongcharoenkamol,[a, b] Prakansi Naksing,[c] Nattayaporn Nimnuan,[c] Thishana Singh,[d] Jaruwan Chatwichien,[e] Prapapan Temkitthawon,[a, b] Chanitsara Sriwattanawarunyoo,[c] Vachira Choommongkol,[f] Puttinan Meepowpan[g, h] and Sutthichat Kerdphon*[b, c, i]

[a] Department of Pharmaceutical Chemistry and Pharmacognosy, Faculty of Pharmaceutical Science, Naresuan University, Phitsanulok 65000, Thailand.
[b] Center of Excellence in Cannabis Research, Faculty of Pharmaceutical Sciences, Naresuan University, Phitsanulok, 65000, Thailand
[c] Department of Chemistry, Faculty of Science, Naresuan University, Phitsanulok 65000, Thailand.
[d] School of Chemistry and Physics, University of Kwazulu-Natal, Private Bag X54001, Durban, 4000, South Africa.
[e] Program in Chemical Sciences, Chulabhorn Graduate Institute, Chulabhorn Royal Academy, Bangkok 10210, Thailand.
[f] Department of Chemistry, Faculty of Science, Maejo University, Chiang Mai 50290, Thailand.
[g] Department of Chemistry, Faculty of Science, Chiang Mai University, Chiang Mai 50200, Thailand.
[h] Center of Excellence in Material Science and Technology, Chiang Mai University, Chiang Mai 50200, Thailand.
[i] Center of Excellence in Petroleum, Petrochemicals and Advanced Materials, Faculty of Science, Naresuan University, Phitsanulok 65000, Thailand.

Table of contents

1. General methods S-1
2. Procedure for synthesis of quinazolinone S-2
3. NMR spectra of the quinazolinone products S-5
4. Acetylcholineesterese (AChE) activity S-22

1. General methods

The Duran® Culture Tube with PBT screw caps were dried in the oven (120 °C) over night and cooled to room temperature. All aminobenzamide substrates and aldehdyes were purchased from commercially available sources. The thin-layer chromatography (TLC) was performed on aluminum plates coated with Kieselgel 60 (0.20 mm, UV 254) and visualized under ultraviolet light. Silica gel (70 – 230 mesh) was purchased from Silicycle. ^{1}HNMR spectra were recorded at 300 MHz in DMSO-d_6 and referenced internally to the residual DMSO-d_6 signal 2.50 ppm). ^{13}CNMR spectra were recorded at 75 MHz in DMSO-d_6 and referenced to the central peak of DMSO-d_6 (39.92ppm). Chemical shifts were reported in ppm (δ scale) and coupling constants (*J*) were reported in Hertz

(Hz). High Performance Microwave Digestion System, Milestone MA182 ETHOS UP with PTFE vessel.

2. Procedure for synthesis of quinazolinone

A mixture of *o*-aminobenzamide (0.5 mmol), aldehdyes (2.5 mmol), CuI (20 mol%) and Cs_2CO_3 (0.75 mmol) under oxygen atmosphere were mixed in Duran® Culture Tube with PBT screw caps. The culture tube was inserted to PTFE vessel including 15 mL of purified water, then was kept in the microwave reactor (High Performance Microwave Digestion System, Milestone ETHOS UP) at 130 °C for 2 hours. After that the reaction mixture was cooled down to room temperature, the crude mixture was purified by dry loading silica gel column chromatography (gradient elution: 20%-70% v/v EtOAc/hexane) to yield a corresponding quinazolinone products. The spectroscopic data of the known quinazolinones were agreement with the previously reported data, the references as shown in below.

Compound	Quizanolione	Yield	Spectroscopic data
**3a**	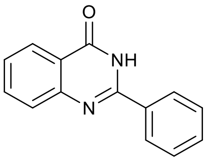	90	Sahoo, S.; Pal, S., *The Journal of Organic Chemistry* **2021,** 86(24), 18067-18080.
**3b**	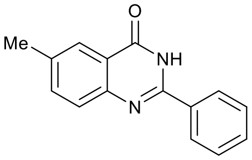	56	Parua, S.; Das, S.; Sikari, R.; Sinha, S.; Paul, N. D., *The Journal of Organic Chemistry* **2017,** 82(14), 7165-7175.
**3c**	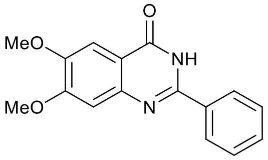	40	Chen, J.; Liang, E.; Shi, J.; We, Y.; Wen, K.; Yao, X.; Tang, X., *RSC Advance* **2021**, 11, 4966-4970.
**3d**	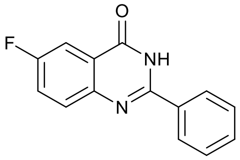	79	Sardar, B.; Jamatia, R.; Samanta, A.; Srimani, D., *The Journal of Organic Chemistry* **2022**, 87(9), 5556-5567.

**3e**	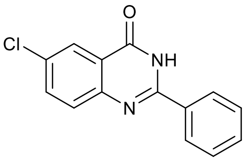	46	Sardar, B.; Jamatia, R.; Samanta, A.; Srimani, D., *The Journal of Organic Chemistry* **2022**, 87(9), 5556-5567.
**3f**	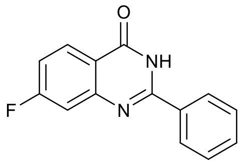	36	Chen, J.; Liang, E.; Shi, J.; We, Y.; Wen, K.; Yao, X.; Tang, X., *RSC Advance* **2021**, 11, 4966-4970.
**3g**	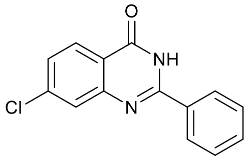	62	Chen, J.; Liang, E.; Shi, J.; We, Y.; Wen, K.; Yao, X.; Tang, X., *RSC Advance* **2021**, 11, 4966-4970.
**3h**	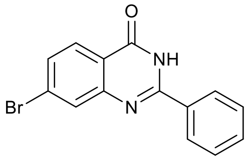	14	Chen, J.; Liang, E.; Shi, J.; We, Y.; Wen, K.; Yao, X.; Tang, X., *RSC Advance* **2021**, 11, 4966-4970.
**3i**	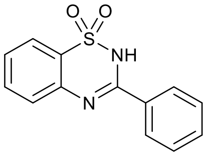	41	Jang, Y.; Lee, S. B.; Hong, J.; Lee, J.; Hong, S., *Organic & Biomolecular Chemistry* **2020**, 18, 5435-5441.
**3j**	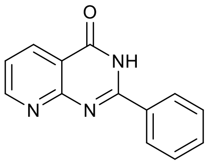	48	Jang, Y.; Lee, S. B.; Hong, J.; Lee, J.; Hong, S., *Organic & Biomolecular Chemistry* **2020**, 18, 5435-5441.
**4a**	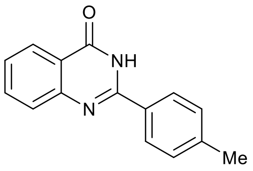	83	Parua, S.; Das, S.; Sikari, R.; Sinha, S.; Paul, N. D., *The Journal of Organic Chemistry* **2017,** 82(14), 7165-7175.
**4b**	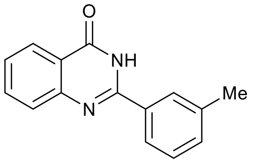	56	Parua, S.; Das, S.; Sikari, R.; Sinha, S.; Paul, N. D., *The Journal of Organic Chemistry* **2017,** 82(14), 7165-7175.
**4c**	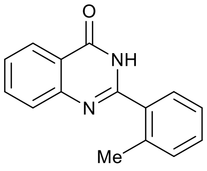	54	Parua, S.; Das, S.; Sikari, R.; Sinha, S.; Paul, N. D., *The Journal of Organic Chemistry* **2017,** 82(14), 7165-7175.

**4d**	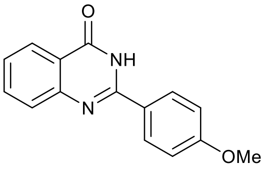	60	Sardar, B.; Jamatia, R.; Samanta, A.; Srimani, D., *The Journal of Organic Chemistry* **2022**, 87(9), 5556-5567.
**4e**	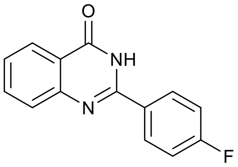	58	Parua, S.; Das, S.; Sikari, R.; Sinha, S.; Paul, N. D., *The Journal of Organic Chemistry* **2017,** 82(14), 7165-7175.
**4f**	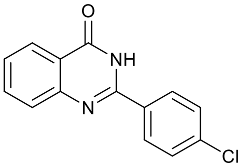	observed	Sahoo, S.; Pal, S., *The Journal of Organic Chemistry* **2021,** 86(24), 18067-18080.
**4g**	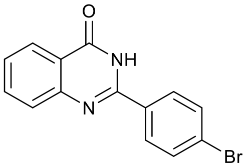	observed	Parua, S.; Das, S.; Sikari, R.; Sinha, S.; Paul, N. D., *The Journal of Organic Chemistry* **2017,** 82(14), 7165-7175.
**4h**	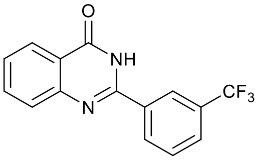	73	Laha, J. K.; Tummalapalli, K. S. S.; Nair, A.; Patel, N., *The Journal of Organic Chemistry* **2015,** 80(22), 11351-11359.
**4i**	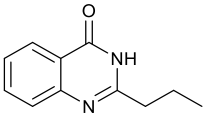	92	Tain, X.; Song, L.; Li, E.; Wang, Q.; Yu, W.; Chang, J., *RSC Advance*, **2015**, 5, 62194-62201.
**4j**	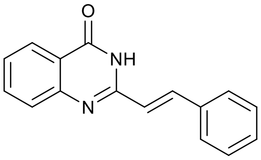	55	Sahoo, S.; Pal, S., *The Journal of Organic Chemistry* **2021,** 86(24), 18067-18080.

3. NMR spectra of the quinazolnone products

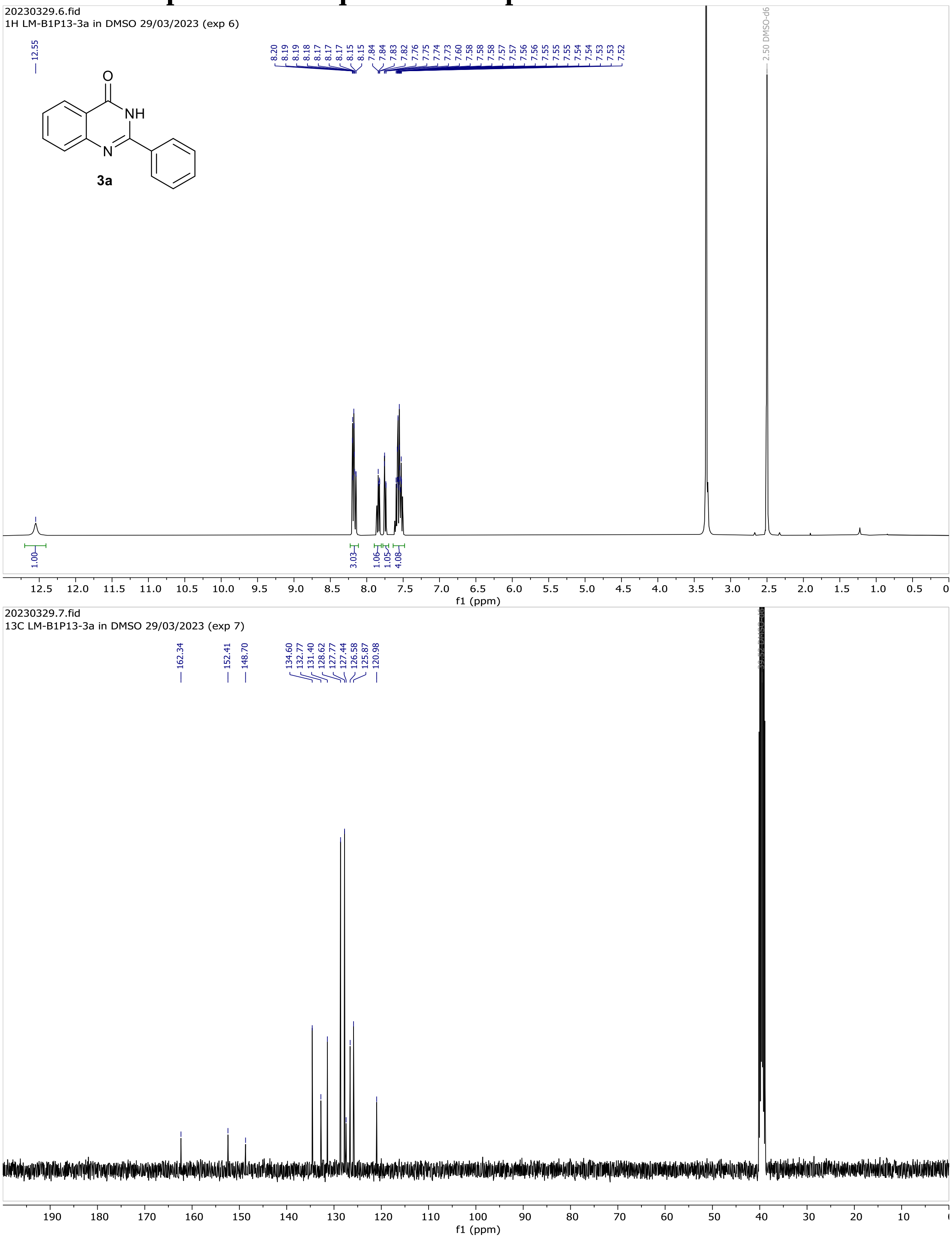

Figure 1 ^{1}H NMR and ^{13}C NMR spectra of **3a**

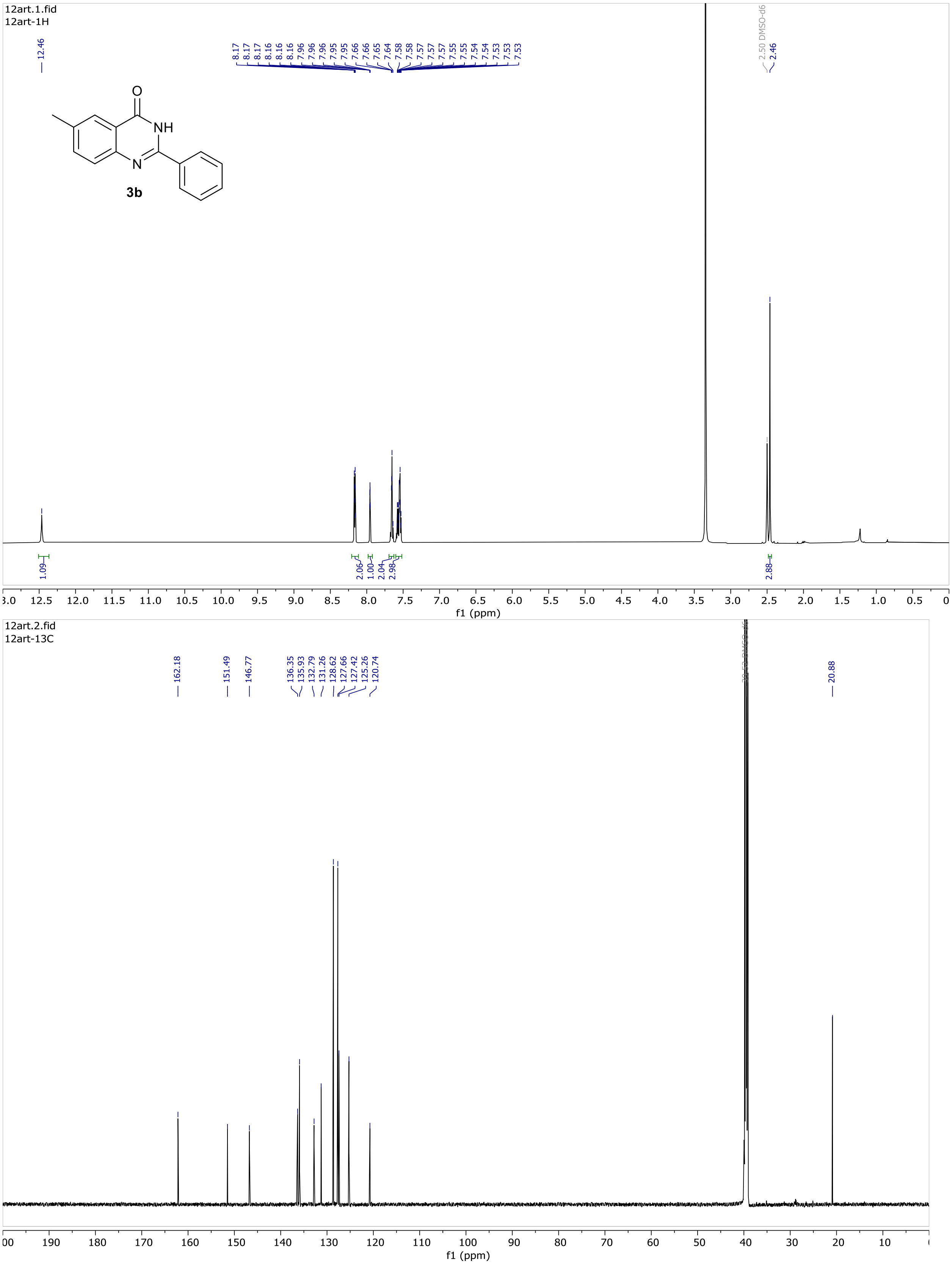

Figure 2 ^{1}H NMR and ^{13}C NMR spectra of **3b**

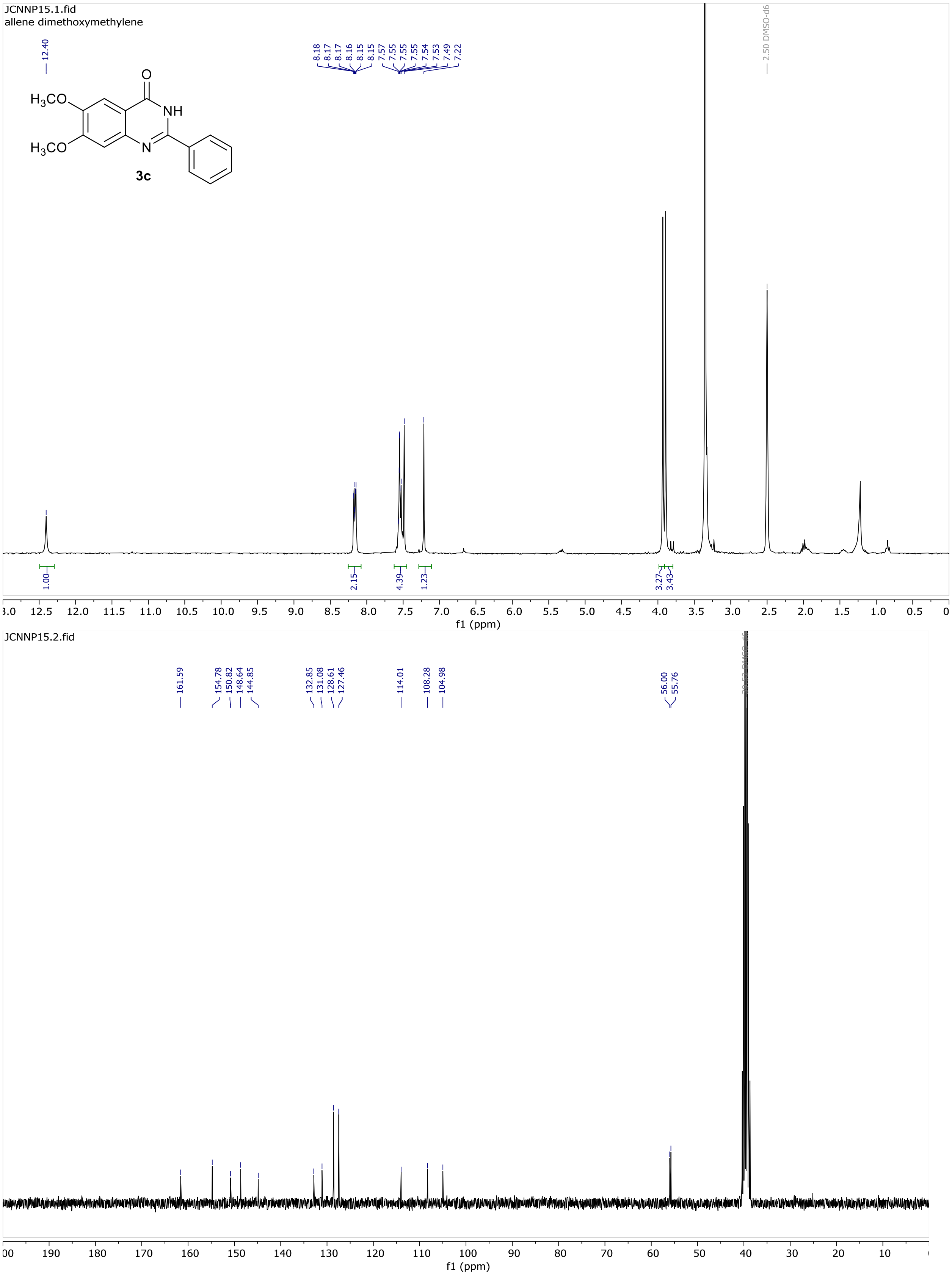

Figure 3 ^{1}H NMR and ^{13}C NMR spectra of **3c**

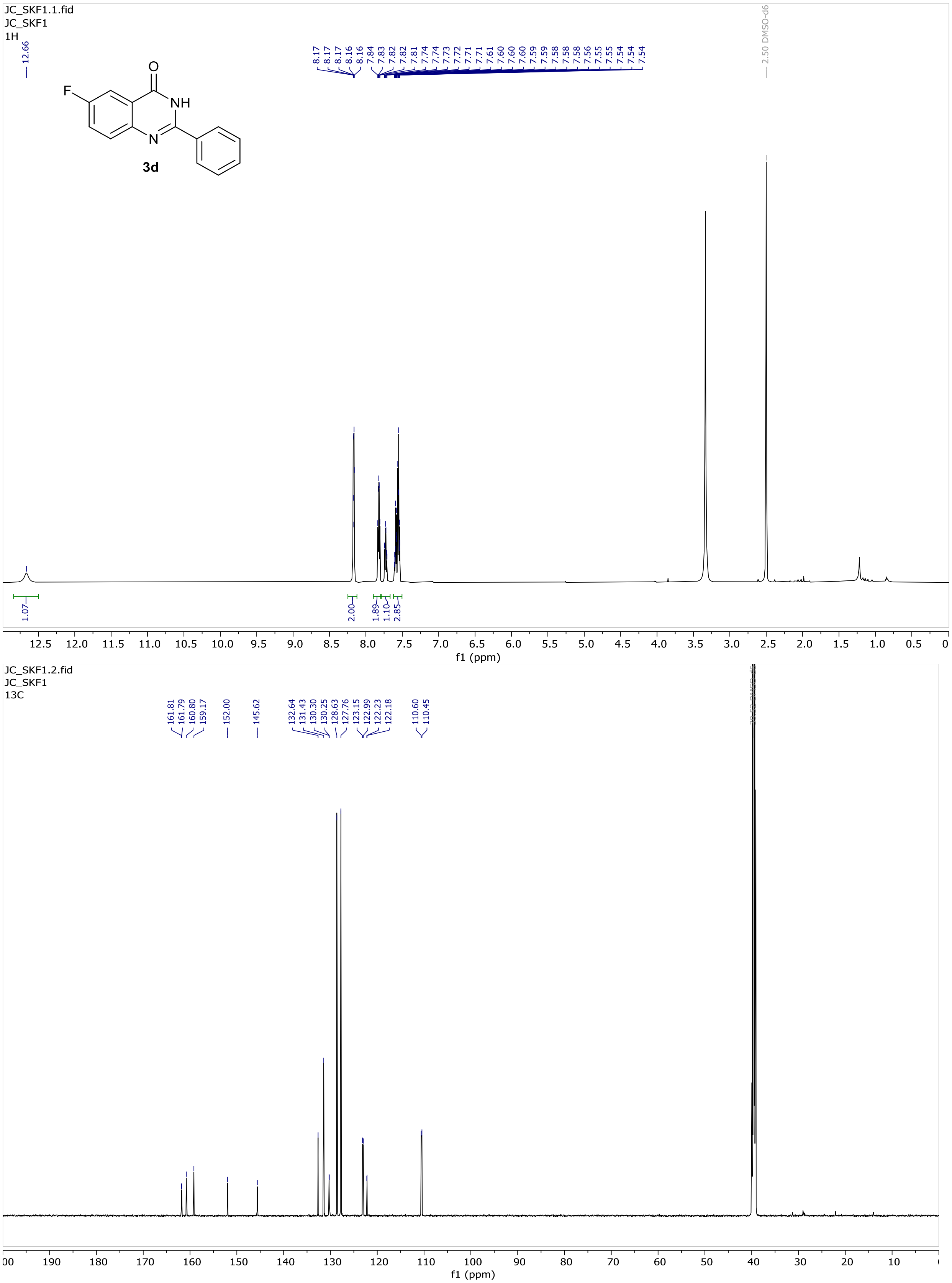

Figure 4 ^{1}H NMR and ^{13}C NMR spectra of **3d**

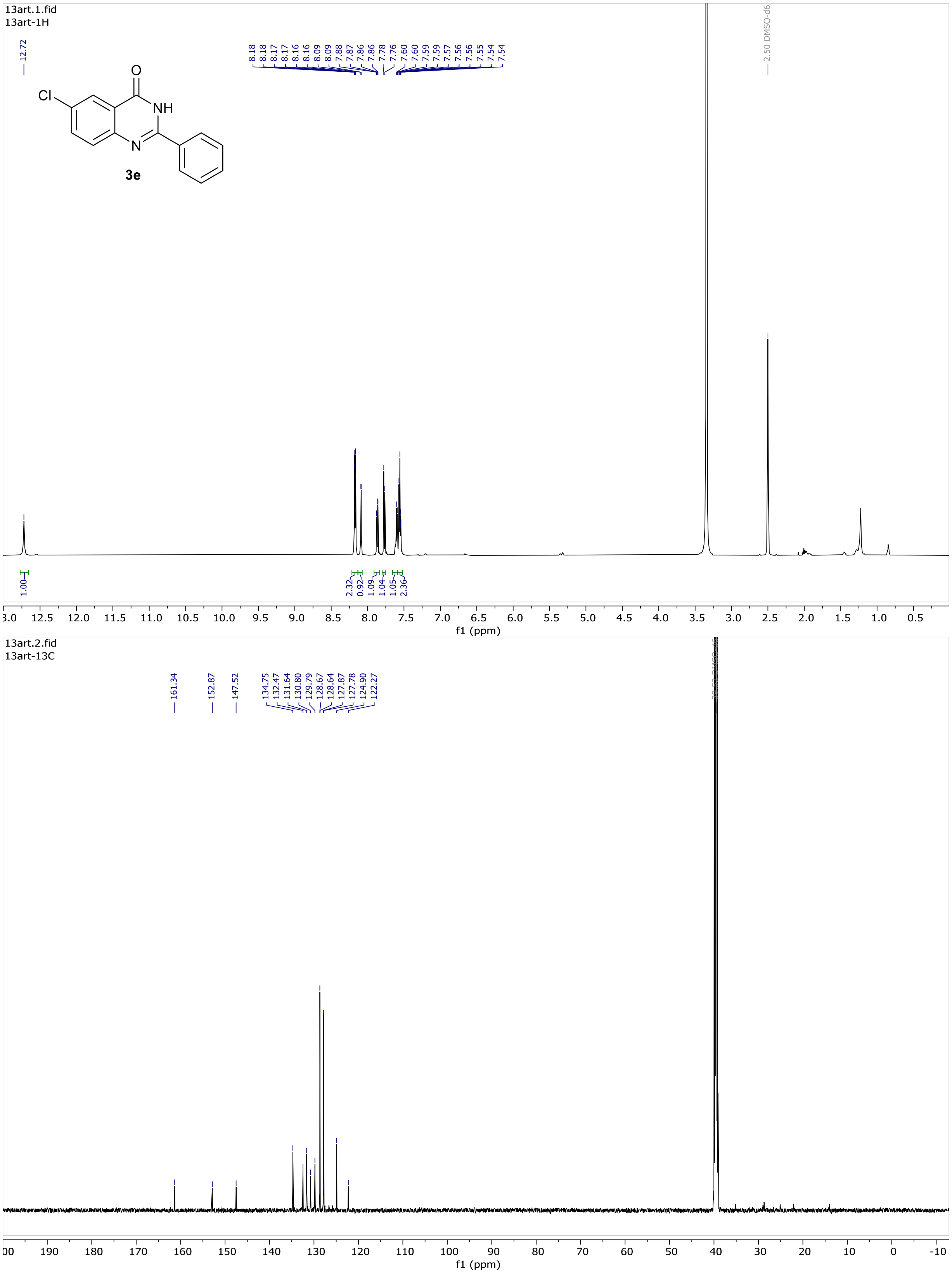

Figure 5 ^{1}H NMR and ^{13}C NMR spectra of **3e**

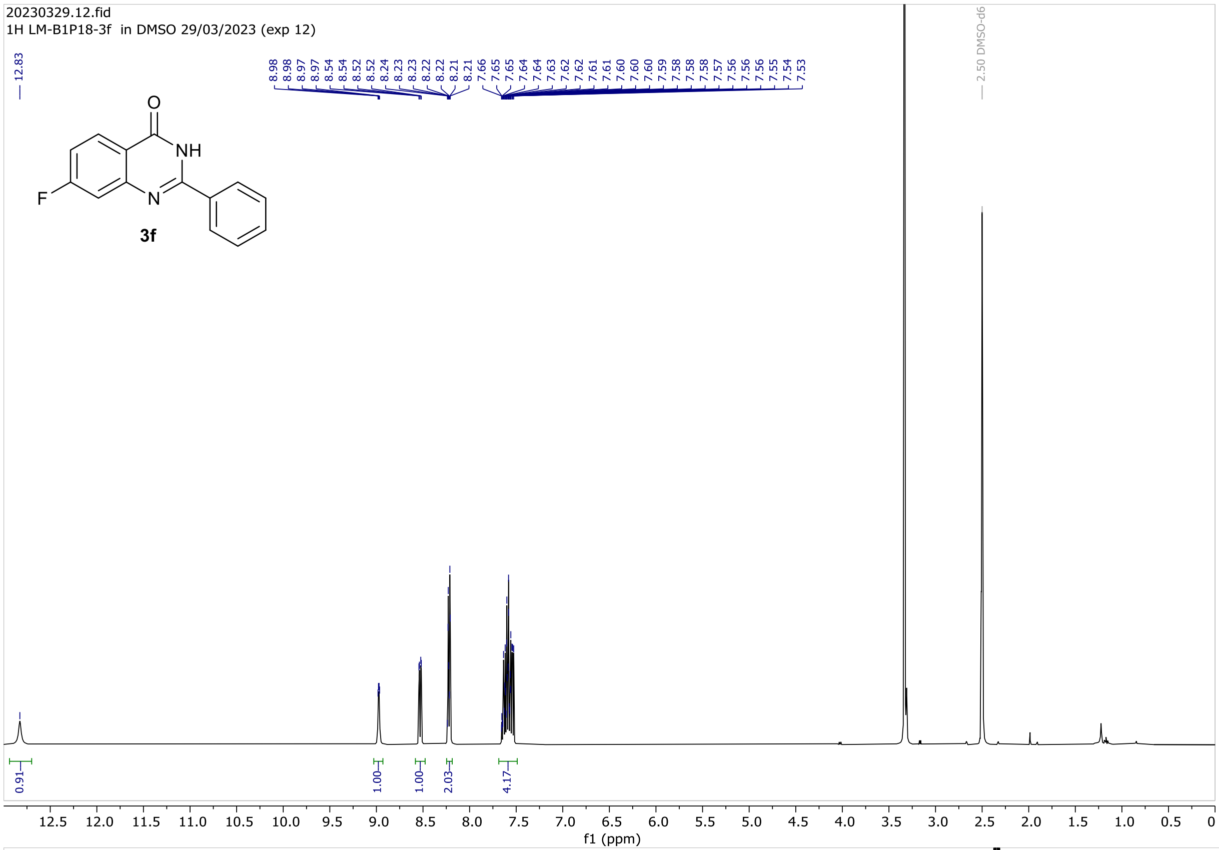

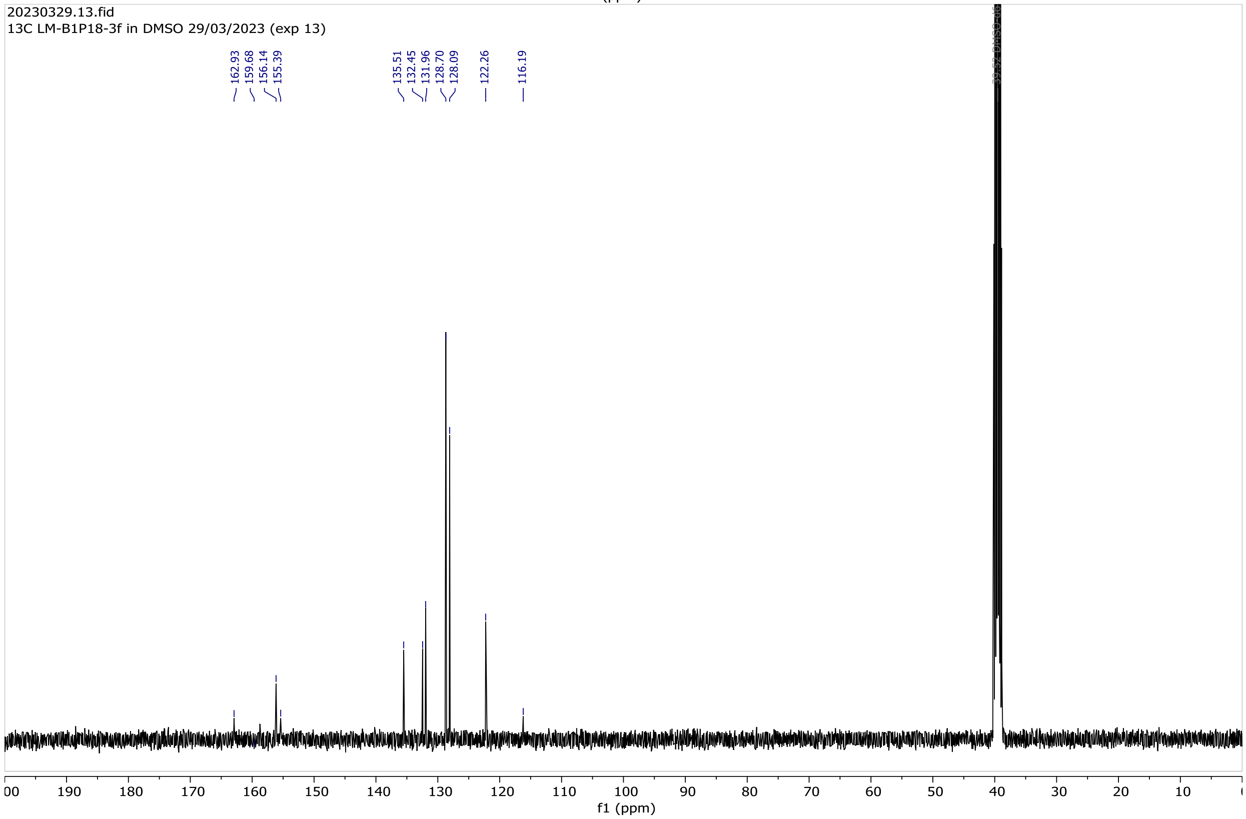

Figure 6 ^{1}H NMR and ^{13}C NMR spectra of **3f**

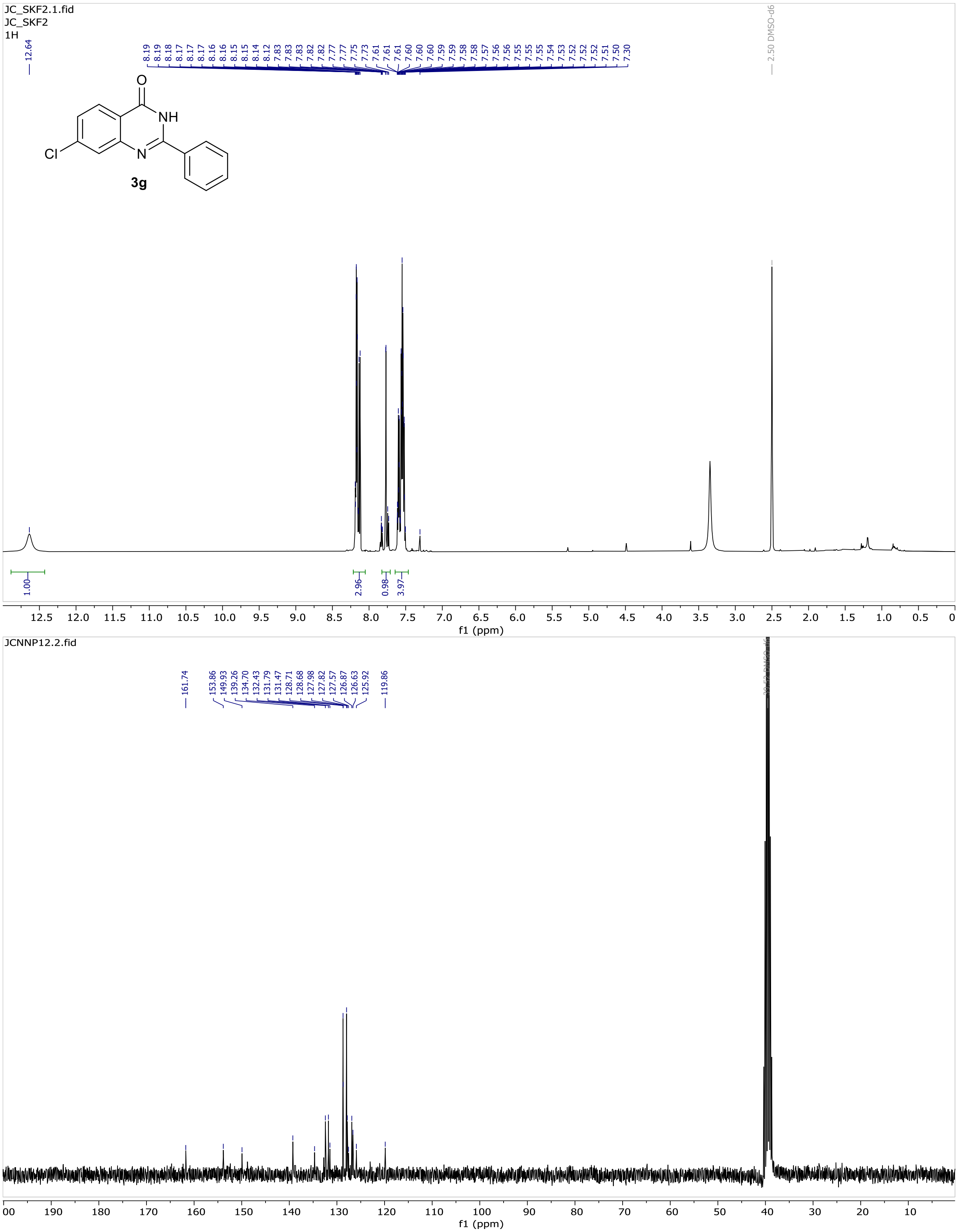

Figure 7 ^{1}H NMR and ^{13}C NMR spectra of **3g**

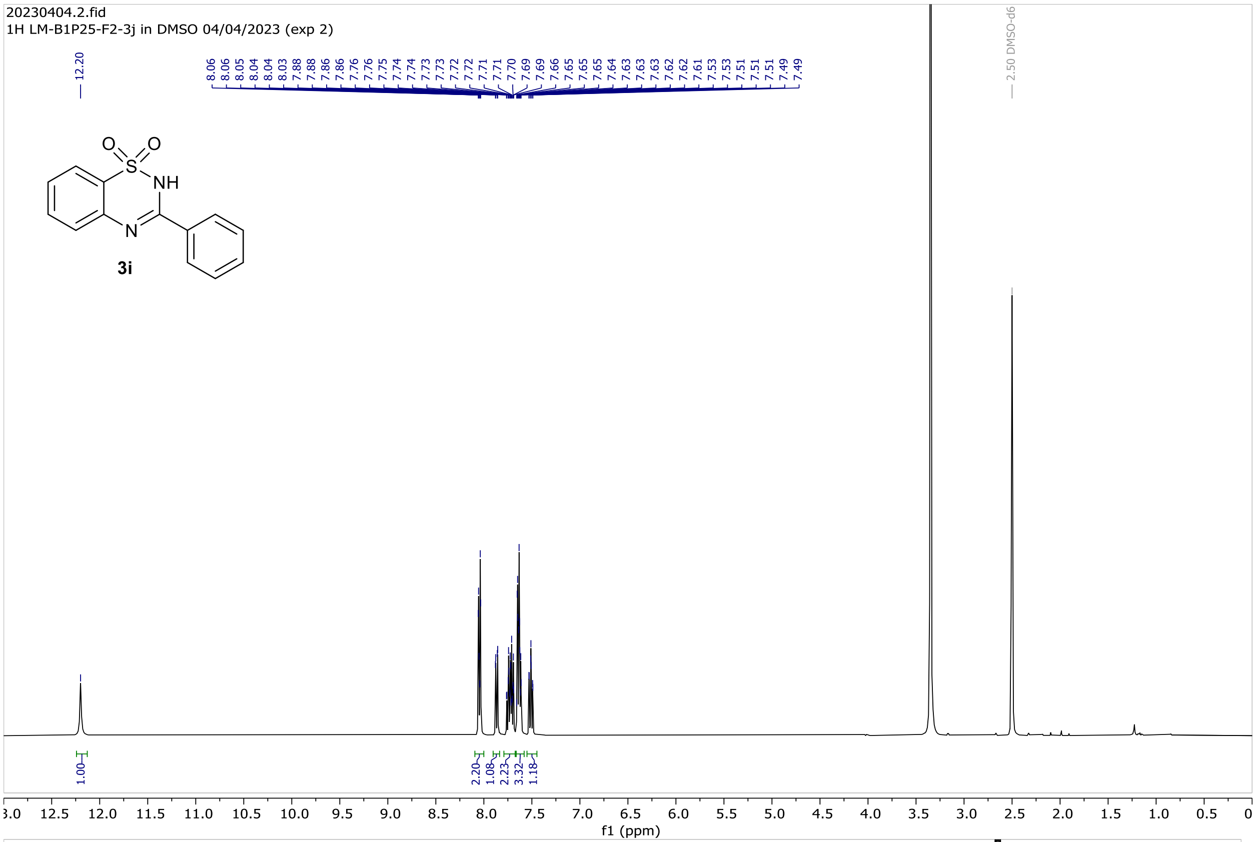

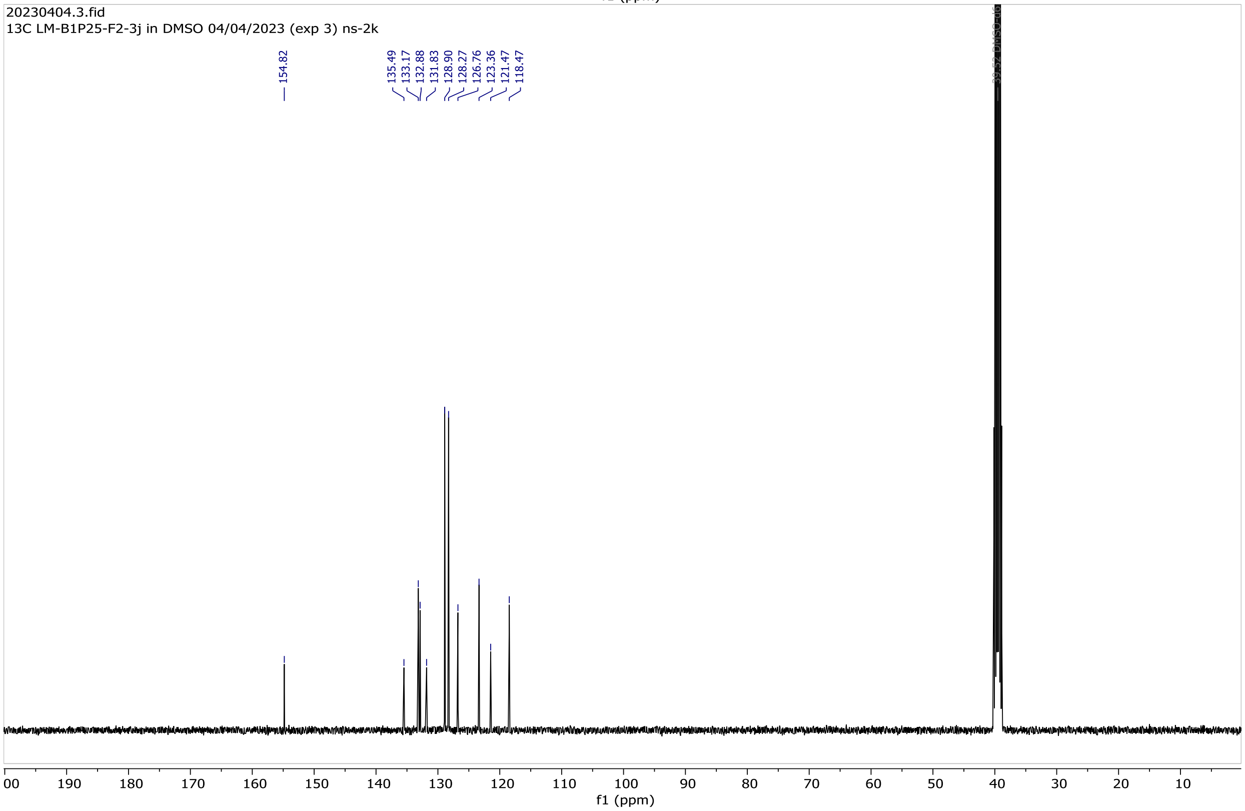

Figure 8 ^{1}H NMR and ^{13}C NMR spectra of **3i**

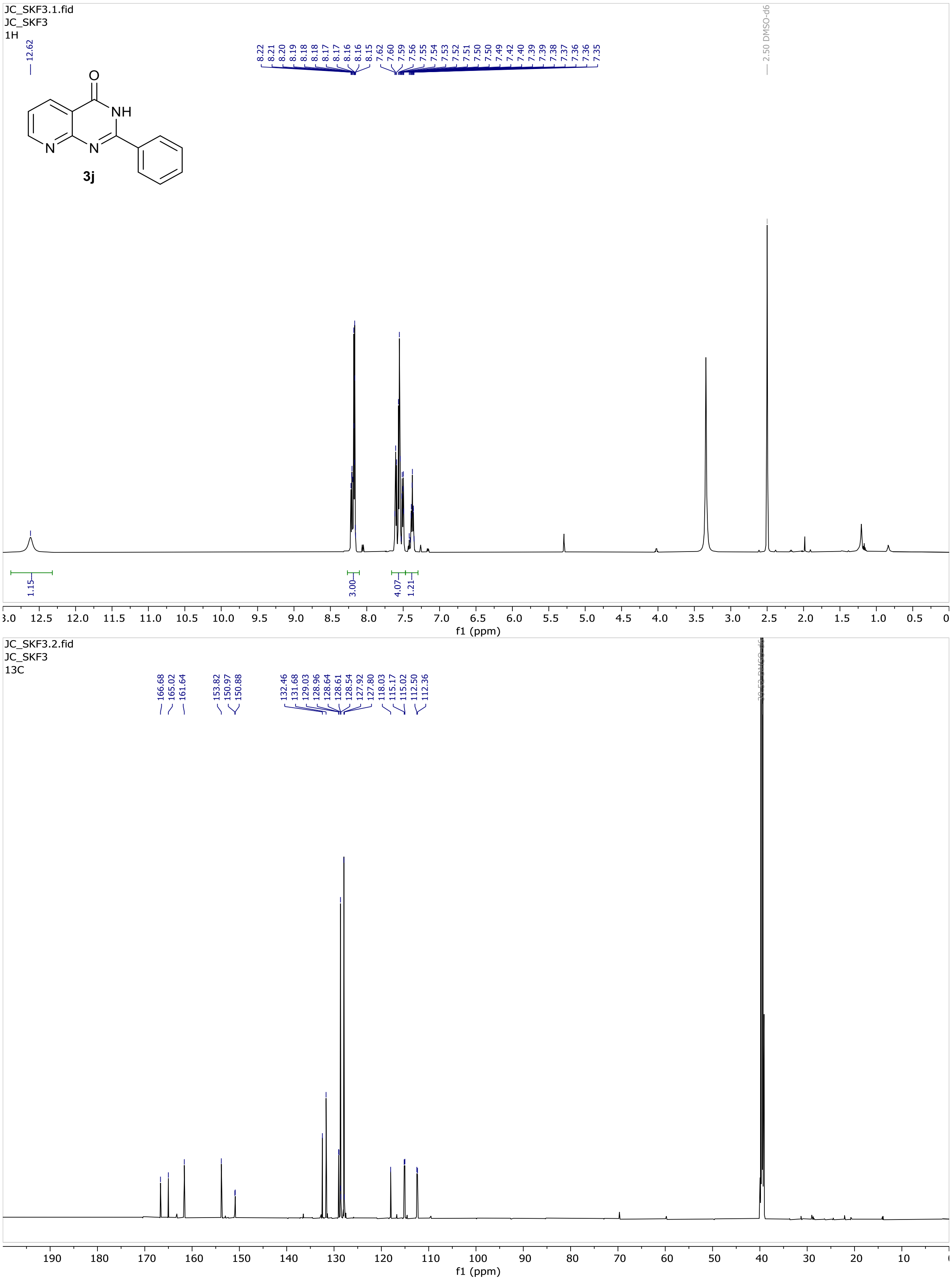

Figure 9 ^{1}H NMR and ^{13}C NMR spectra of **3j**

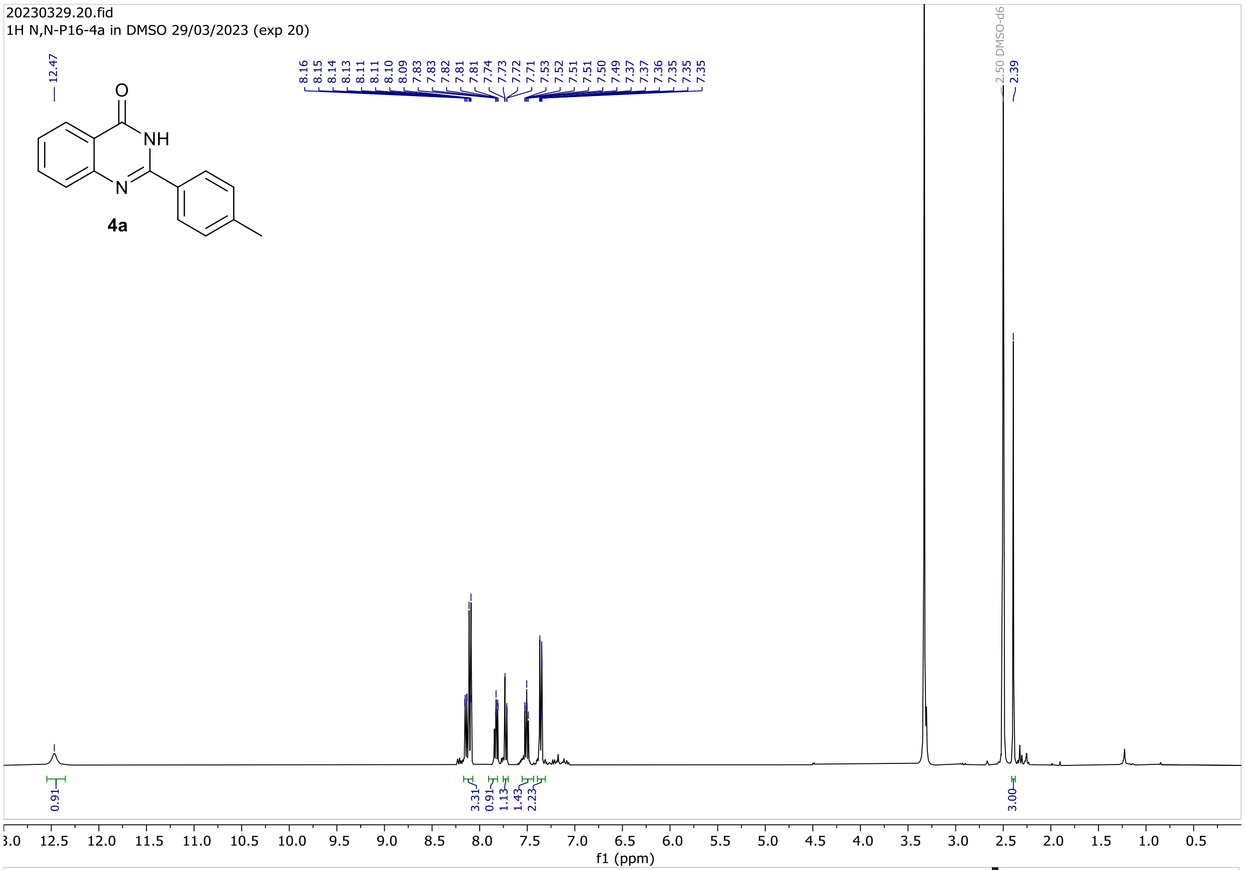

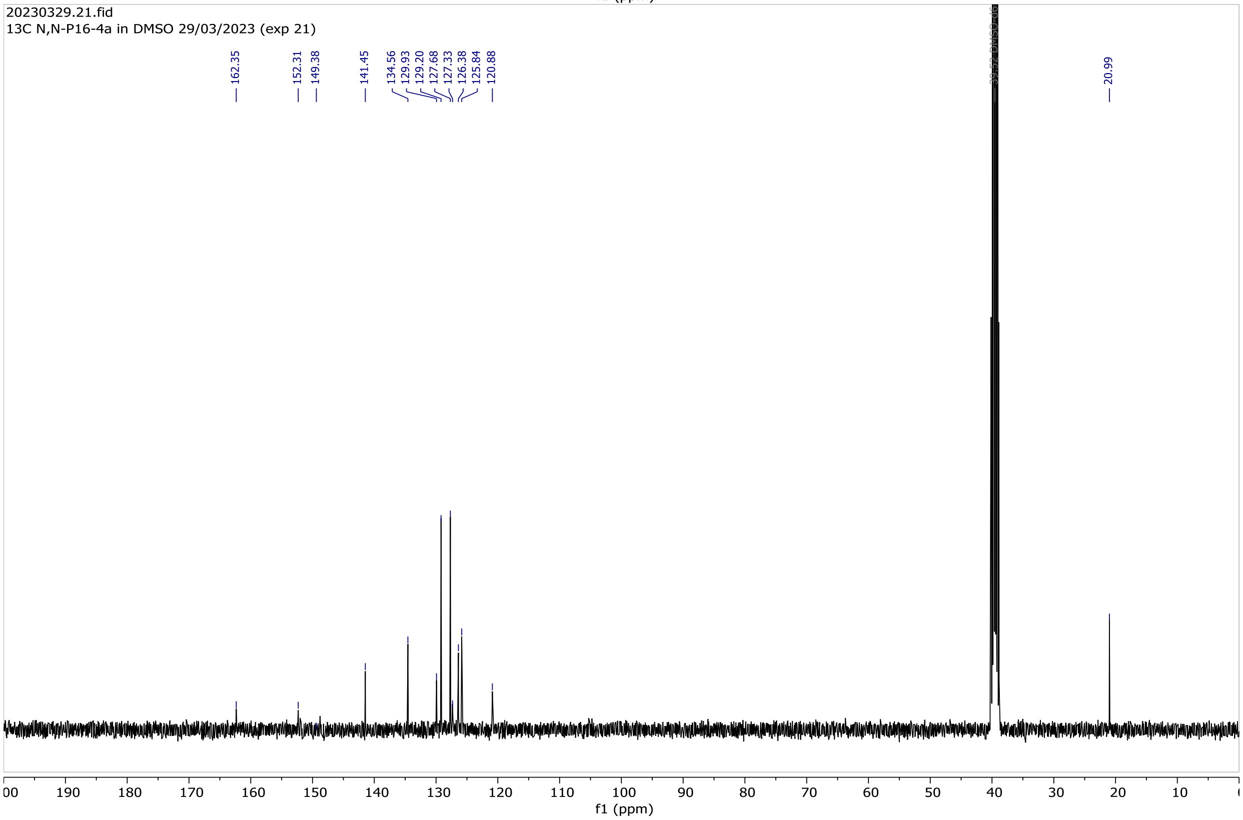

Figure 10 ^{1}H NMR and ^{13}C NMR spectra of **4a**

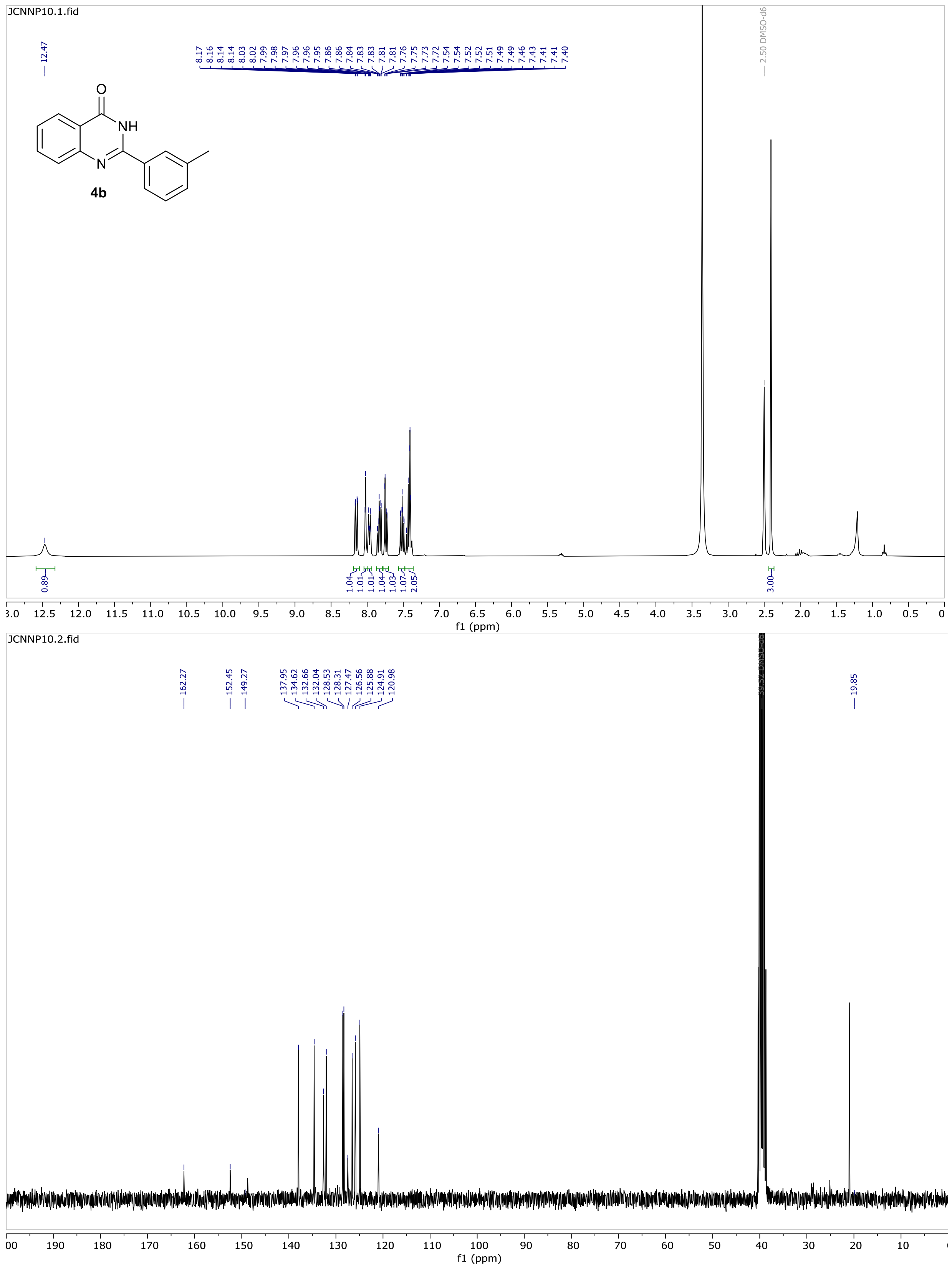

Figure 11 ^{1}H NMR and ^{13}C NMR spectra of **4b**

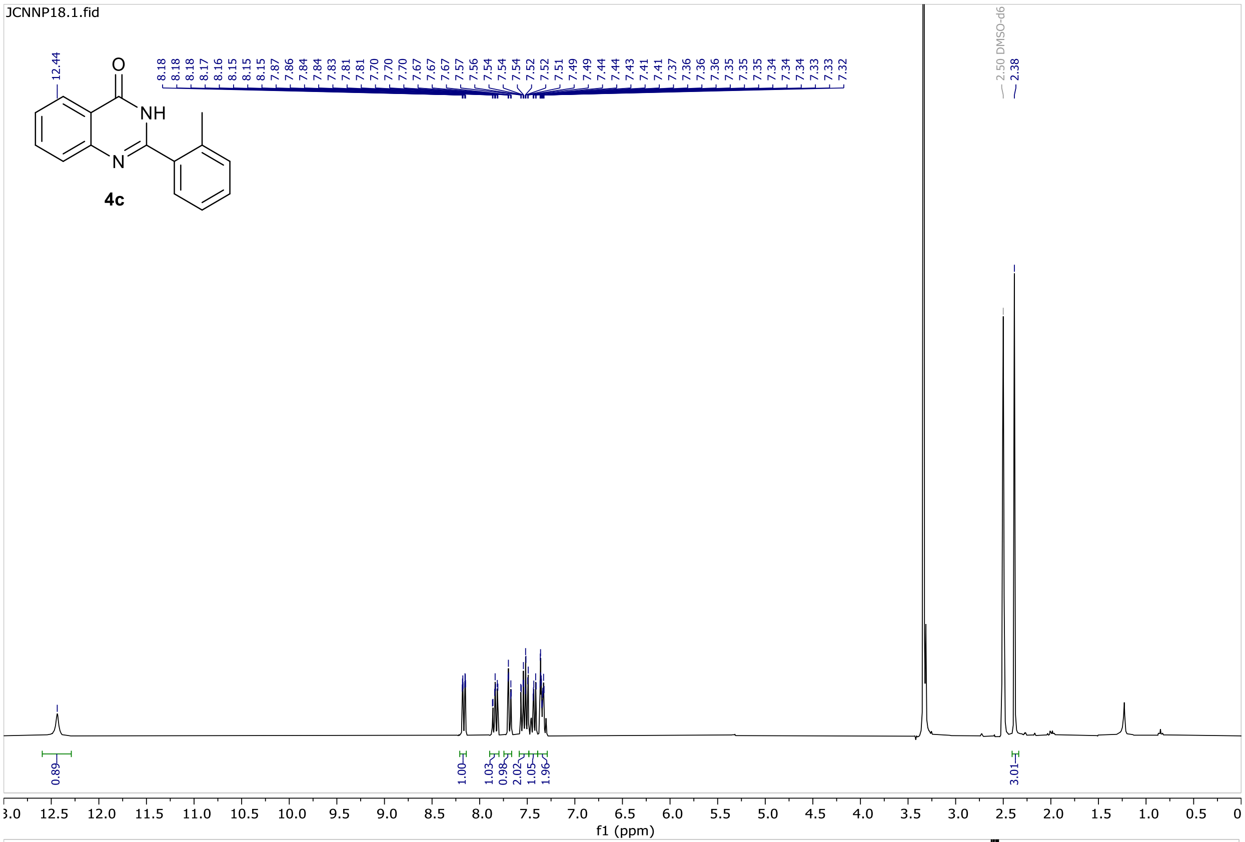

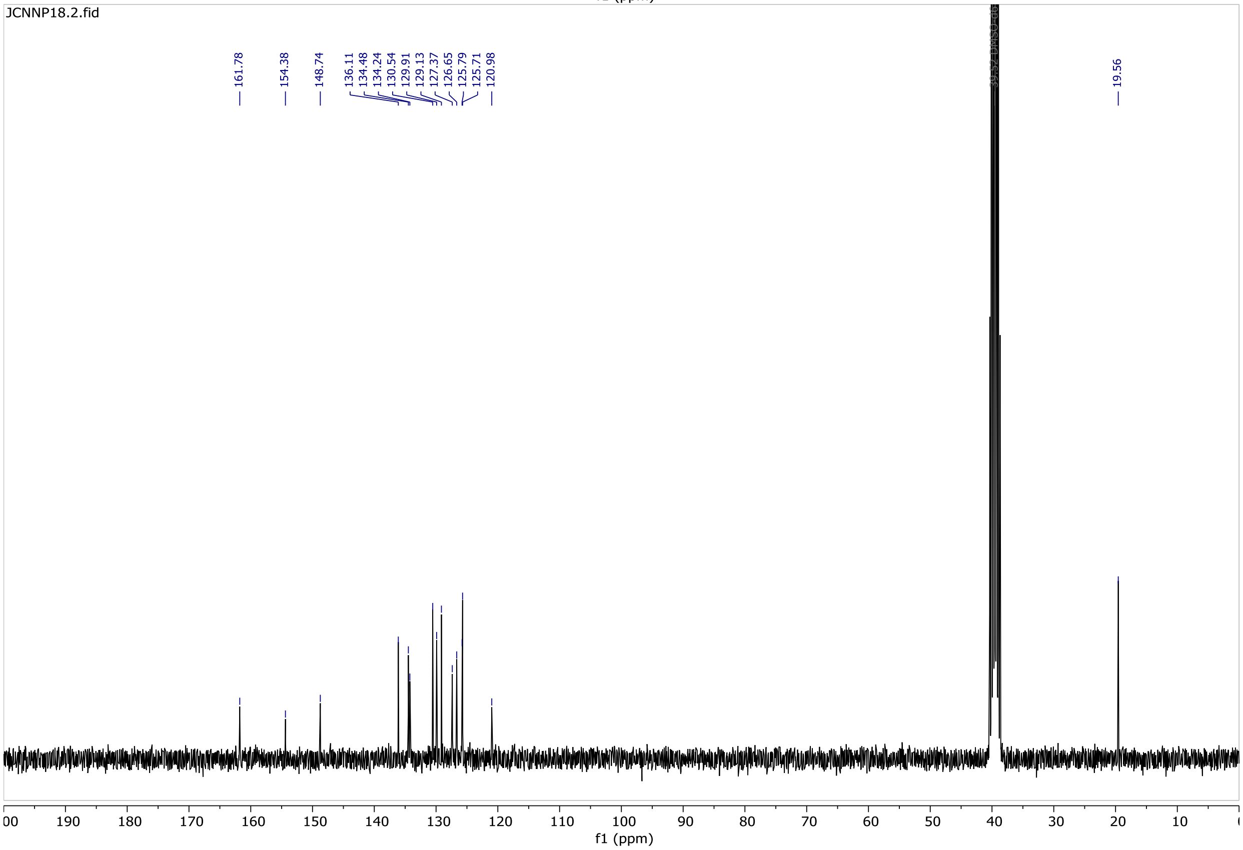

Figure 12 ^{1}H NMR and ^{13}C NMR spectra of **4c**

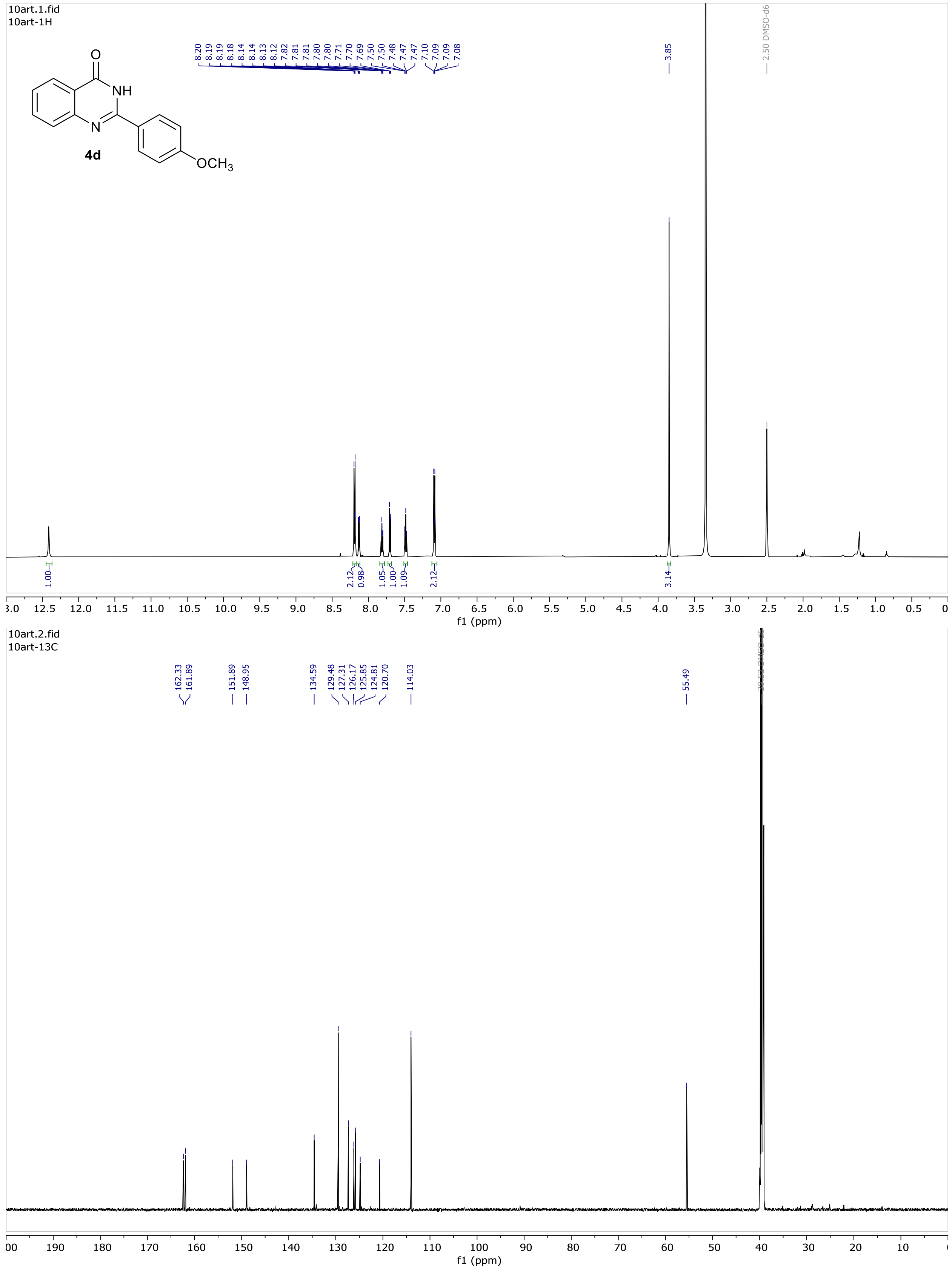

Figure 13 ^{1}H NMR and ^{13}C NMR spectra of **4d**

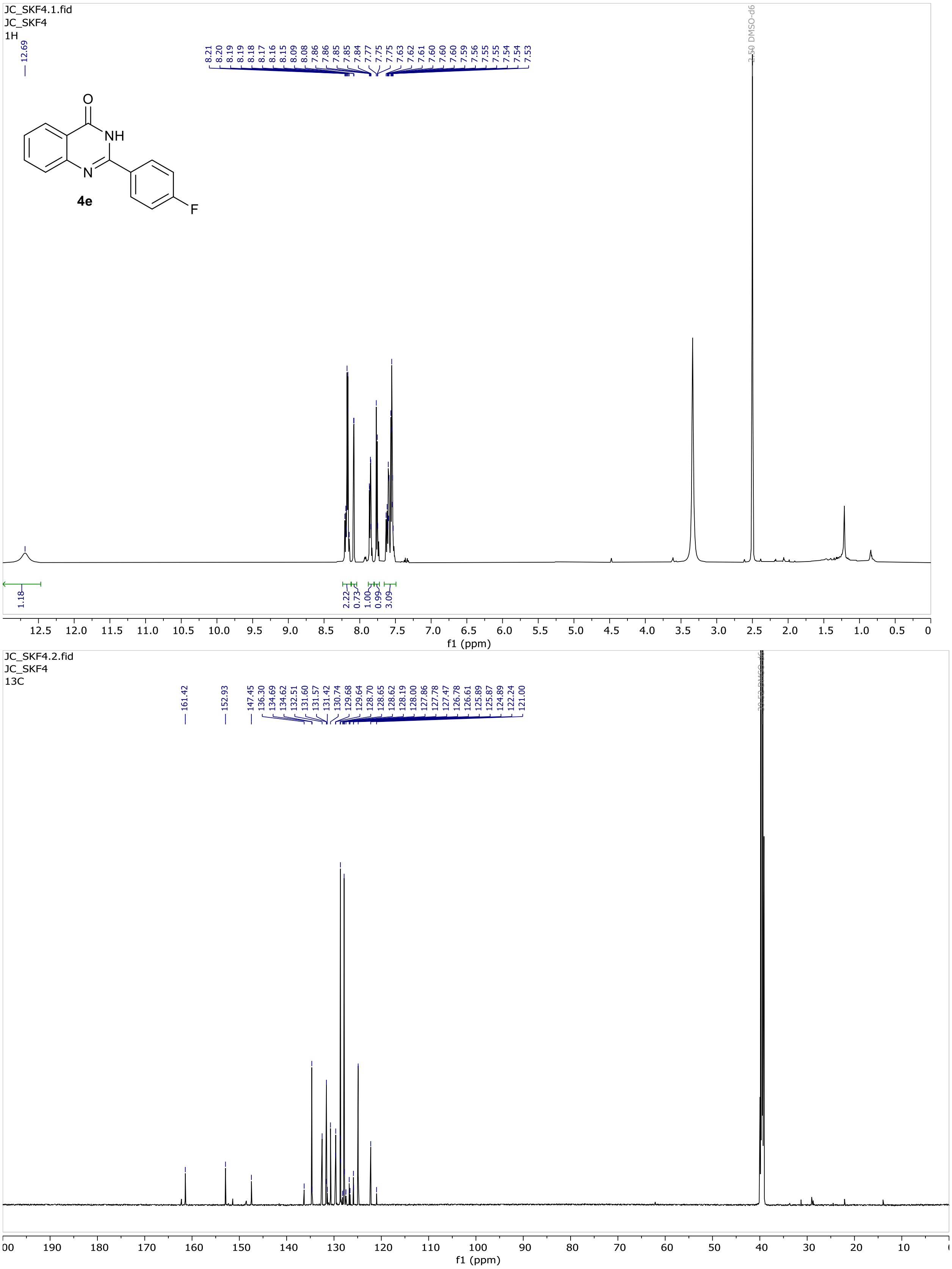

Figure 14 ^{1}H NMR and ^{13}C NMR spectra of **4e**

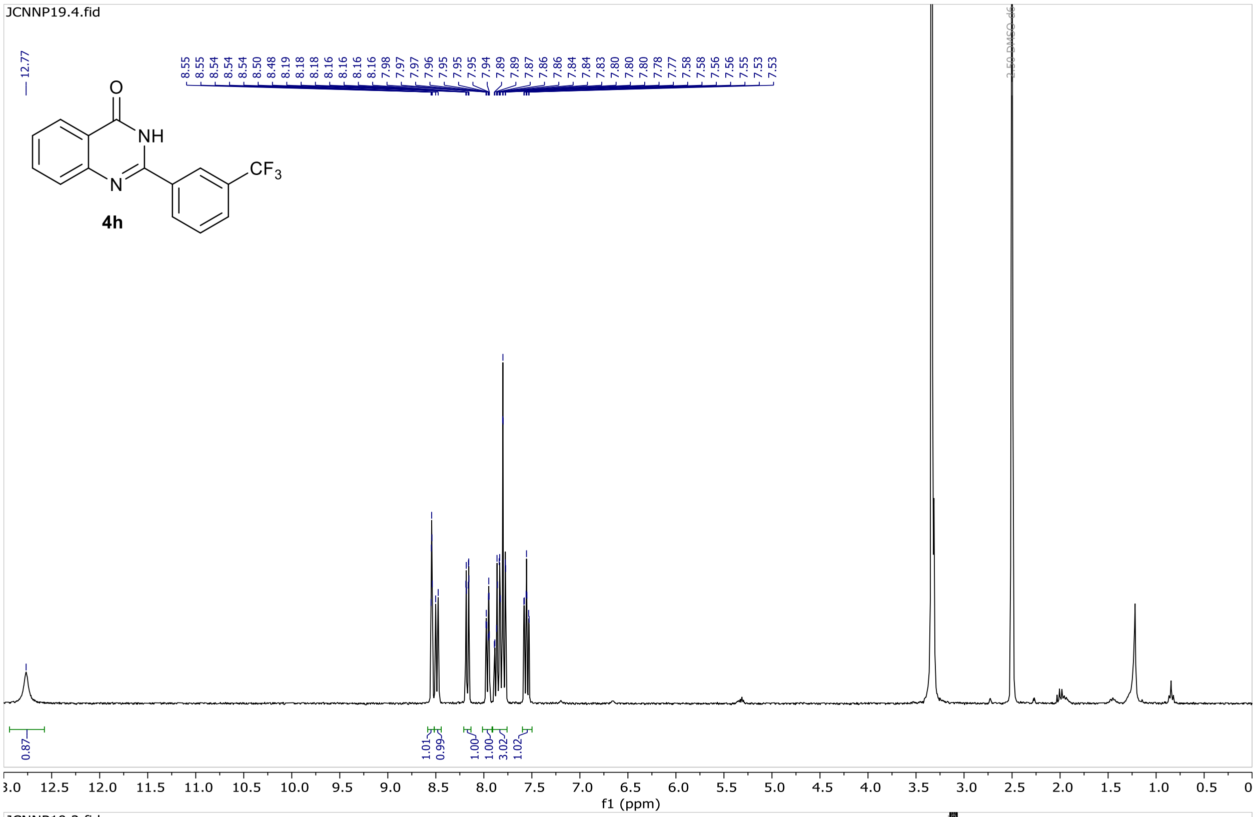

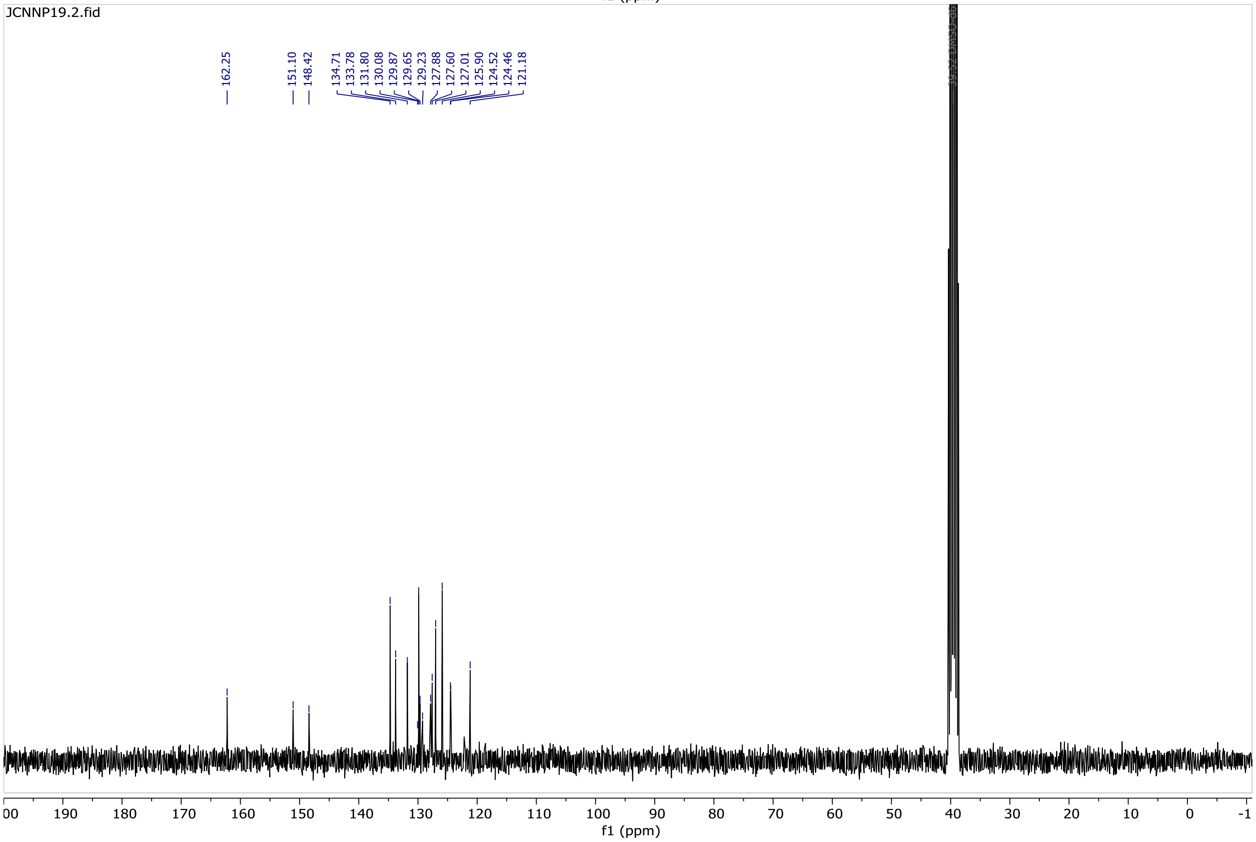

Figure 15 ^{1}H NMR and ^{13}C NMR spectra of **4h**

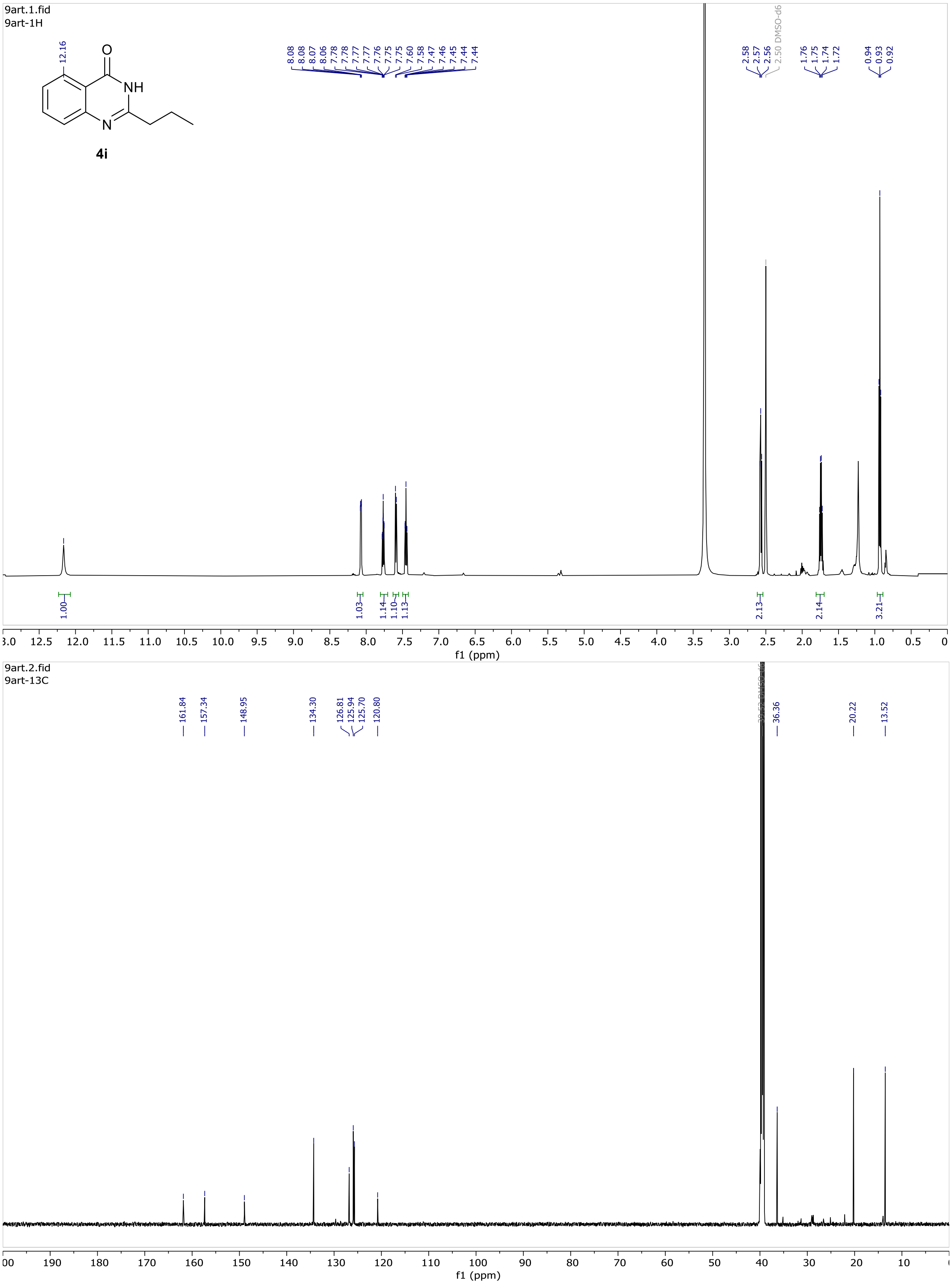

Figure 16 ^{1}H NMR and ^{13}C NMR spectra of **4i**

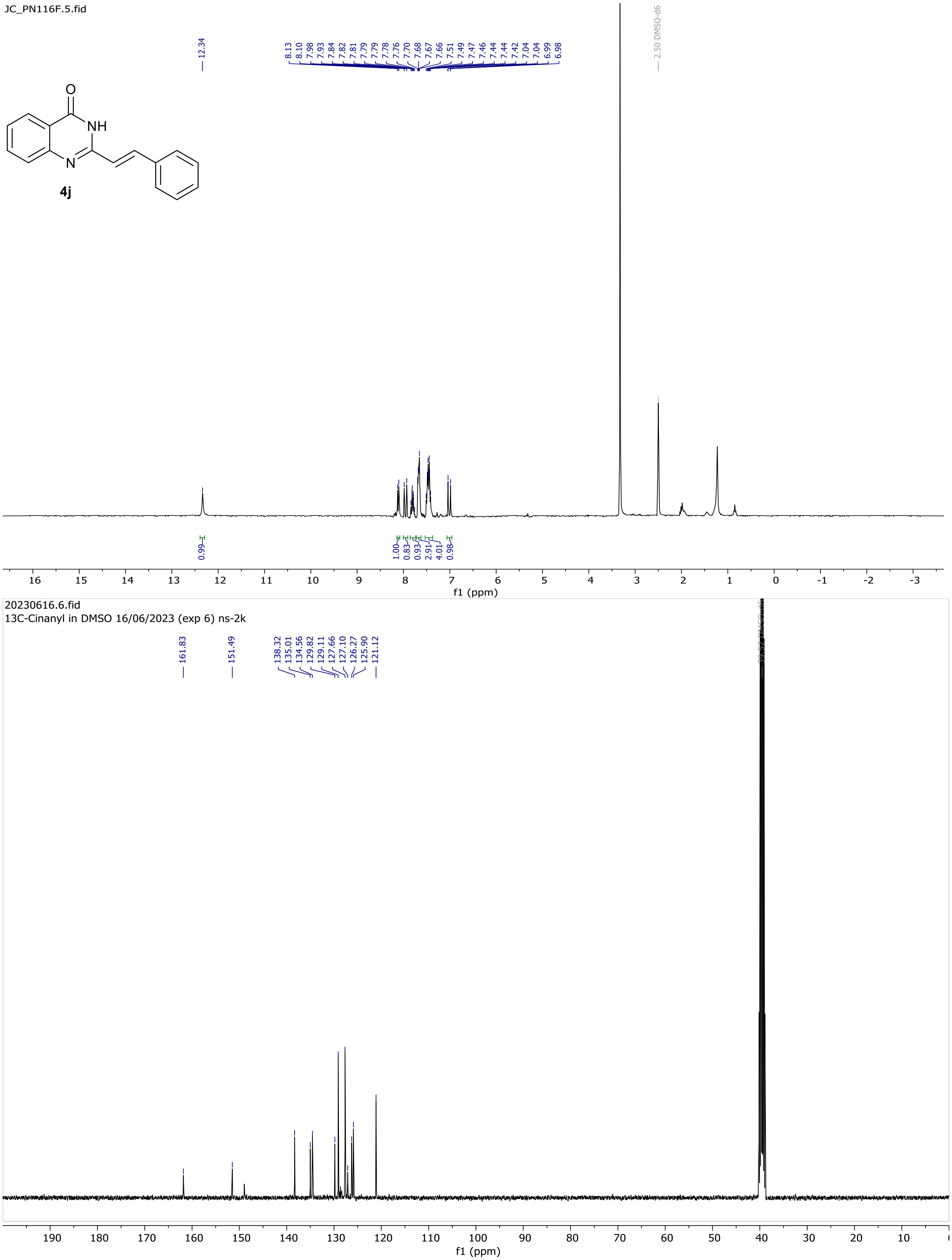

Figure 17 ^{1}H NMR and ^{13}C NMR spectra of **4j**

4. Acetylcholineesterese (AChE) activity

3 mM Ellman's reagent in Tris/HCl pH 8 125 µl, 1.5 mM acetylthiocholine iodide in water 25 µl and Tris/HCl pH 8 buffer 50 µl and sample 25 µl were mixed in 96-well plate. The mixture was added with AChE solution in 1 mg/ml BSA 25 µl then immediately measured at 405 nm every 50 second for 5 min.

$$\%\text{inhibition} = \frac{(\text{Mean velocity}_{\text{control}} - \text{Mean velocity}_{\text{sample}}) \times 100}{\text{Mean velocity}_{\text{control}}}$$
